# ADP-dependent glucokinase as a novel onco-target for haematological malignancies

**DOI:** 10.1038/s41598-020-70014-0

**Published:** 2020-08-12

**Authors:** Amol Tandon, Jana Birkenhagen, Deepthi Nagalla, Stefan Kölker, Sven Wolfgang Sauer

**Affiliations:** 1 Division of Child Neurology and Metabolic Diseases, University Children’s Hospital Heidelberg, Im Neuenheimer Feld 430, 69120 Heidelberg, Germany; 2 German Cancer Research Center (DKFZ), 69120 Heidelberg, Germany; 3 Present Address: Department of Biochemistry and Molecular Genetics, University of Alabama at Birmingham, Birmingham, AL 35233 USA

**Keywords:** Biochemistry, Cancer, Cell biology, Immunology, Molecular biology

## Abstract

Warburg effect or aerobic glycolysis provides selective growth advantage to aggressive cancers. However, targeting oncogenic regulators of Warburg effect has always been challenging owing to the wide spectrum of roles of these molecules in multitude of cells. In this study, we present ADP-dependent glucokinase (*ADPGK*) as a novel glucose sensor and a potential onco-target in specifically high-proliferating cells in Burkitt’s lymphoma (BL). Previously, we had shown *ADPGK* to play a major role in T-cell activation and induction of Warburg effect. We now report *ADPGK* knock-out Ramos BL cells display abated in vitro and in vivo tumour aggressiveness, via tumour-macrophage co-culture, migration and Zebrafish xenograft studies. We observed perturbed glycolysis and visibly reduced markers of Warburg effect in *ADPGK* knock-out cells, finally leading to apoptosis. We found repression of *MYC* proto-oncogene, and up to four-fold reduction in accumulated mutations in translocated *MYC* in knock-out cells, signifying a successful targeting of the malignancy. Further, the activation induced differentiation capability of knock-out cells was impaired, owing to the inability to cope up with increased energy demands. The effects amplified greatly upon stimulation-based proliferation, thus providing a novel Burkitt’s lymphoma targeting mechanism originating from metabolic catastrophe induced in the cells by removal of *ADPGK*.

## Introduction

The process known as Warburg effect or aerobic glycolysis provides an evolutionary growth advantage to cancer cells and in recent years has become increasingly important as a target for curbing aggressively growing malignancies^1–4^. Burkitt’s lymphoma (BL), a malignancy of germinal centre B-cells, is the fastest growing human cancer^5^ and owing its growth to one of the most widely studied oncogenes and regulator of glycolysis, *MYC*, provides a perfect model to study Warburg effect^6,7^. The translocation t(8,14)(q24,q32) is the characteristic genetic hallmark of Burkitt’s lymphoma and is identified in almost all patient biopsies. This translocation brings *MYC* proto-oncogene on one allele into proximity with the immunoglobulin locus (H/L (heavy/light) chain) and leaving the other allele as wild-type *MYC*^8–11^. As a result, a constitutive overexpression of *MYC* is observed in Burkitt’s lymphoma resulting in dysregulation of *MYC* expression due to the influence of heavy transcriptional activity of this locus^9^. Additionally, stimulation (T-cell dependent /independent) driven differentiation of B-cells is marked by an initial activation phase characterized by high proliferation and Warburg like upregulation of metabolism and growth, and subsequent differentiation to plasma/memory cells^12–16^. These stages of proliferation and differentiation represent ideal scenarios to analyse the regulation of metabolic activity of a fast-growing cancer under activated and quiescent states.

**Figure. 1:**
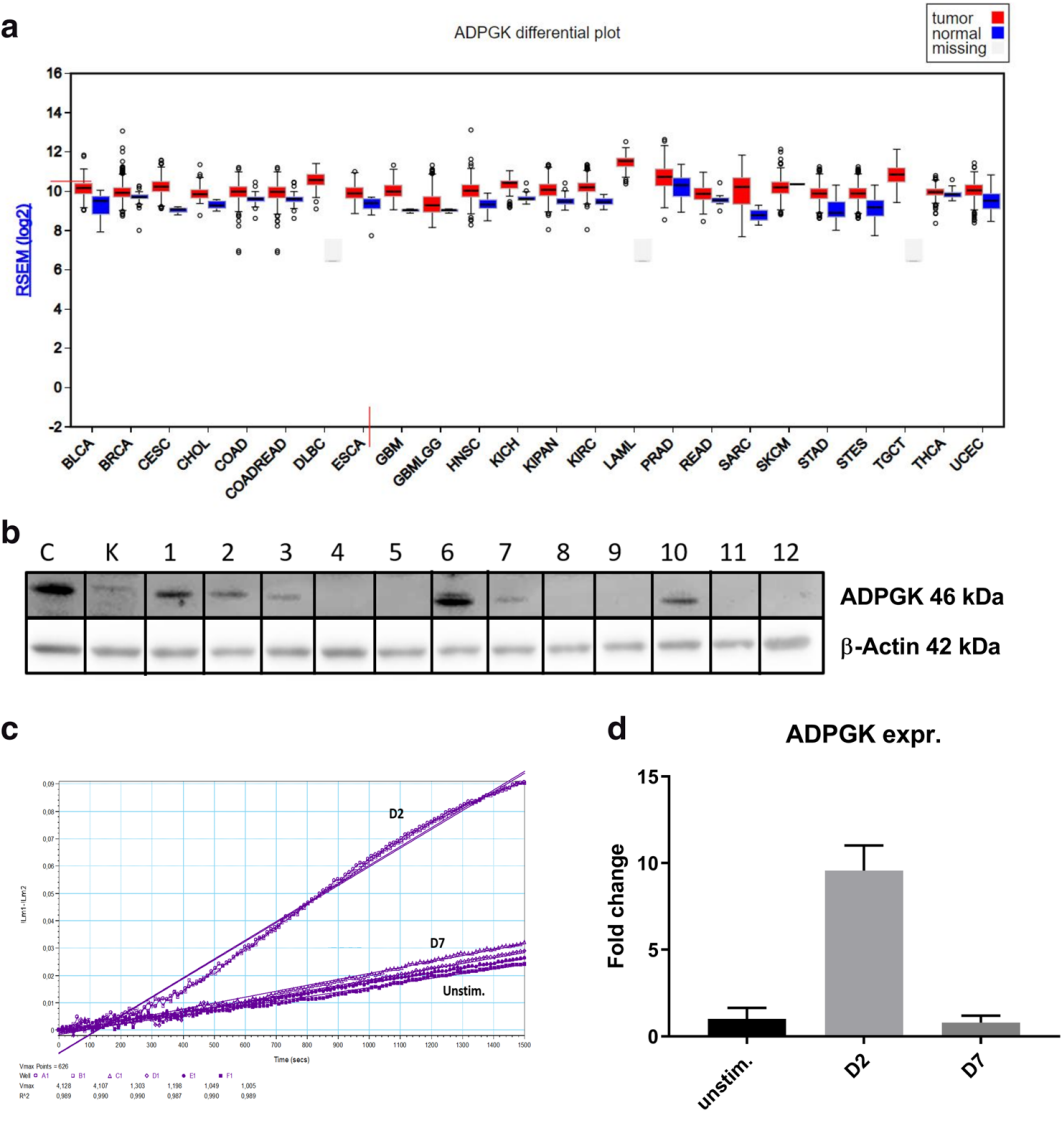
ADPGK activity and expression upon stimulation. (**a**) Expression data for ADPGK in normal and tumour samples in the TCGA (The Cancer Genome Atlas) FireBrowse expression viewer. Tumour expression- red blocks; Normal tissue expression- blue blocks (**b**) *ADPGK* knock-outs were generated via CRISPR/Cas9 technology targeting exon-2 of *ADPGK*. ADPGK (ADP dependent glucokinase) knock-out selection via western blot using 46 kDa band as reference. β-actin is the loading control. Lanes 1–12 depict 12 analysed clones. Lanes 4 and 8 were selected as KO-1 and KO-2 respectively. Ramos BL (Burkitt's lymphoma) cells were stimulated with 50 ng/ml PMA (phorbol-12 myristate 13-acetate) for a period of seven days and analysed at D2 (two days post stimulation) and D7 (seven days post stimulation). (**c**) Enzyme kinetics of ADPGK at D2, D7 and unstim. (unstimulated) states. X-axis represents time in seconds and y-axis is difference in absorbance between 340 and 400 nm. (**d**) Change in ADPGK expression upon stimulation at D2, D7 and unstimulated states using RT-qPCR. Data is representative of three individual experiments. Error bars represent + /- s.e.m. C is positive control and K negative control for ADPGK. TCGA cancer nomenclature is available at https://gdc.cancer.gov/resources-tcga-users/tcga-code-tables/tcga-study-abbreviations.

In this study, we tried to decipher the metabolic phenotype of Ramos BL cells and their potential to differentiate into Plasma cells in the presence of an important regulator of immune metabolism, ADP-dependent glucokinase (ADPGK). ADPGK is known as a regulator of Warburg effect and has been recently shown to play an important role in T-cell activation and induction of glycolytic phenotype via regulation of N- and O-glycosylation by our lab^17,18^. ADPGK is highly expressed in immune cells of both myeloid and lymphoid lineages and use of ADP instead of ATP by the enzyme for priming glucose hints at its role in nutrient deprived and hypoxic conditions, such as those prevalent in tumour growth, where ATP is available in lean amounts^17,19,20^. A broader role for ADPGK across different malignancies could be seen from its expression in normal and tumour cells, as shown in Fig. 1a.

Therefore, we assessed the growth, differentiation status and various markers for tumour aggressiveness in Ramos BL cells and their *ADPGK* knock-out counterparts, upon activation with a known protein kinase-C (PKC) based inducer of B-cell activation, phorbol 12-myristate 13-acetate (PMA)^21–26^. Hence, we hypothesized that knock-out of *ADPGK* from Ramos BL cells will induce a metabolic catastrophe in these cells, affecting the tumour aggressiveness of these cells in vitro and in vivo in zebrafish model. The knock-out also proposed to stall the activation mediated differentiation of these cells and thereby providing a novel regulator of two mutually complementary, but aerobic glycolysis dependent pathways, malignancy and differentiation.

## Results

### Generation of ADPGK knock-out with CRISPR/Cas9

ADPGK knockouts were generated in Ramos BL cells (Burkitt’s lymphoma) using CRISPR/Cas9 technology and analysed via Western blots. Two knockouts were finally selected for further experiments based on loss of 46 kDa ADPGK protein band in western blot (Fig. 1b.) Additionally sequencing confirmed the presence of heterozygous deletion/insertion in one clone (KO-1: 316_317del and 319_320insC) and homozygous four base deletion in the other (KO-2: 314_317del).

### ADPGK expression and enzymatic activity upon B-cell activation

B-cells stimulated with PMA are known to follow an initial course of activation and proliferation followed by differentiation into plasmablasts forming Memory B-cells or Plasma cells^24–26^. A burst of aerobic glycolysis marks the proliferative phase providing necessary energy and metabolites for growth. We wanted to see the expression changes of *ADPGK* in Ramos BL cells upon activation with phorbol 12-myristate 13-acetate (PMA)^21–26^.

Therefore, Ramos BL cells were stimulated with PMA for seven days and we measured *ADPGK* gene expression and enzyme activity at D2 and D7 representing the proliferating and differentiated cells respectively (Fig. 1c, d). The expression of *ADPGK* increased several folds upon stimulation and peaked at D2 where after it decreased until D7 and became even lower than basal levels. (Fig. 1d) ADPGK enzyme activity was on the other hand undetectable in unstimulated cells but displayed significant increase in kinetics at D2 before again becoming undetectable at D7 (Fig. 1c). This depicted the correlation of expression and enzyme activity of ADPGK with proliferating cells and at the same time displaying its near-redundant nature in quiescent cells.

### PMA stimulation induced differentiation is hampered in ADPGK KO cells

**Figure. 2:**
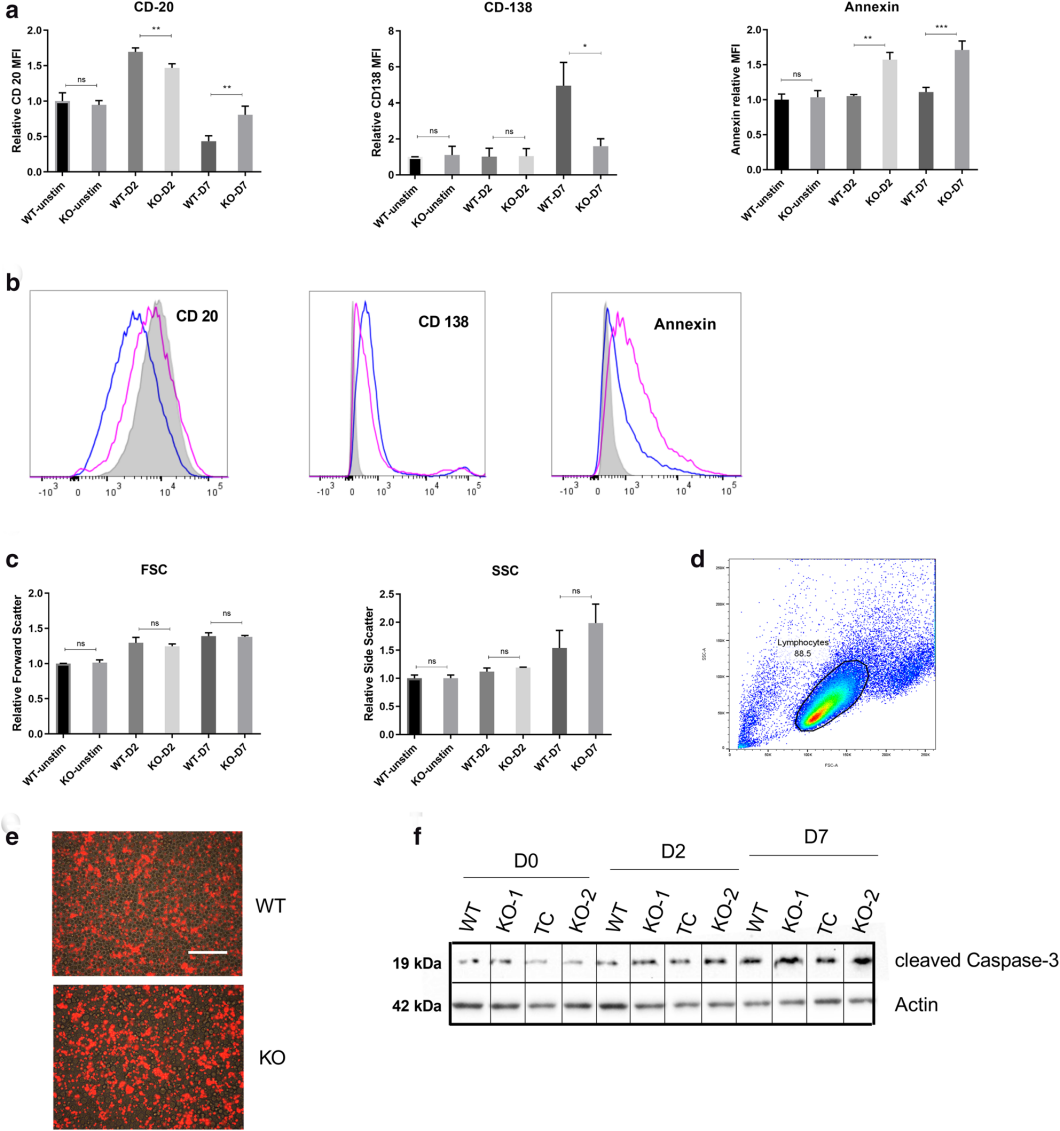
PMA stimulation induced differentiation is hampered in ADPGK KO cells. (**a**) *ADPGK* (KO and WT cells were treated with 50 ng/ml PMA (phorbol-12 myristate 13-acetate) and cultured for seven days under standard conditions. Flow cytometric analysis was performed for cells collected at D0, D2 and D7 (unstimulated, two days stimulated, and seven days) post stimulation. Panel **a** shows the change in Relative Fluorescence Intensity (RFI), from PE (CD20 and CD138) or FITC (Annexin V) tagged fluorescent antibodies. (**b**) The same data represented as histogram of D7 (differentiation phase) fluorescence intensities for respective markers; pink line represents KO cells, blue WT and shaded area as unstimulated WT cells serving as control. (**c**) Forward and side scatter values generated by the flow cytometry experiments on various days for WT and KO cells. (**d**) Gating strategy for excluding cell debris and clumps from analysis with automated area selection for lymphocytes using FlowJo software. Percentage of gated cells used for further analysis is shown by the number in the box. (**e**) Microscopic images of cells collected at D7 and displaying fluorescence for AnnexinV at 10 × magnification. Scale bar corresponds to 200 µm. (**f**) Western blot for Ramos WT and ADPGK KO cells at D0, D2 and D7, probed for cleaved Caspase-3 with β-Actin as loading control. Error bars represent + /- s.e.m of RFI (for CD20, CD138 and AnnexinV) or FSC/SSC values obtained from three independent experiments. *y-axis* for bar-graphs represents mean values of each sample normalized to unstimulated WT cells. *x-axis* in histograms shows fluorescence intensity. 100,000 events were recorded for each flow cytometry experiment. (*p < 0.05; **p < 0.01, ***p < 0.001, calculated using Welch’s t-test for significance).

CD20 and CD138 (Syndecan-1) are important markers for following B-cell differentiation^27,28^. Mature unstimulated B-cells express CD20 but lack CD138 (CD20^+^/CD138^-^). When stimulated by antigens they undergo cell surface changes to activated CD20^+^/CD138^+^ B-cells, and finally differentiate into CD20^-^/CD138^+^ plasma cells or recently classified CD20^-/low^ pre-plasmablasts^26,27,29,30^. Flow cytometry analysis of CD20 on D0, D2, and D7 post stimulation showed a normal differentiation related expression of this molecule on Ramos WT cells (Fig. 2a). With high quantity of CD20 detected in the unstimulated state, the levels of this molecule were upregulated until D2 post PMA stimulation, and exhibited a drastic decrease on D7 reflecting differentiation of WT cells (Fig. 2a, b). The expression of CD138 (Syndecan-1), undetected at D2, increased at D7, though remaining at low levels but signifying a population positive for this plasma cell marker (Fig. 2a). ADPGK KO cells exhibited nearly equal concentration of CD20 in the unstimulated state as the WT cells and the levels of this marker were stable until D2. On D7, the KO cells exhibited low CD20 expression, but levels were still much higher than WT cells. CD138 was slightly upregulated at D7 but overall levels of CD138 remained almost at half the values for WT cells, signifying a reduced capacity of these cells to differentiate into plasma cells (Fig. 2a, b).

**Figure.3:**
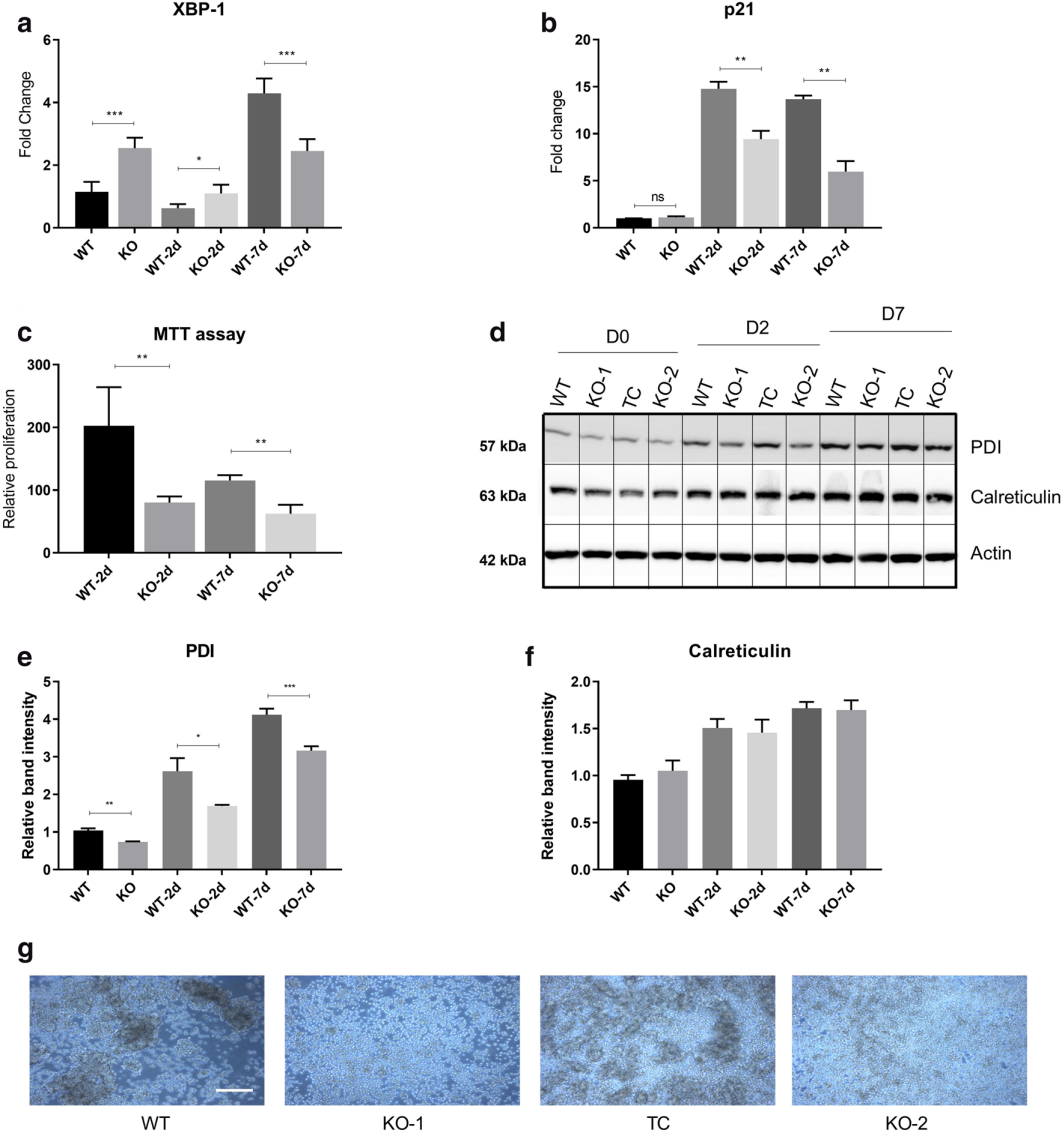
ER stress-based differentiation markers are more expressed in Ramos WT cells. ADPGK KO and Ramos WT cells, treated with PMA and cultured for seven days under standard conditions were analysed via western blot and RT-qPCR at D0, D2 and D7 post stimulation. (**a**) Results of RT-qPCR performed for detecting ratio of spliced:unspliced *XBP1* transcript in ADPGK KO and Ramos WT cells at different conditions. y-axis represents fold change in expression of all samples normalized to unstimulated WT cells. (**b**) RT-qPCR analysis for cell-cycle regulator p21 (*CDKN1A*) in Ramos WT and ADPGK KO cells at different PMA stimulation time points (**c**) MTT assay to analyse the proliferation rate of ADPGK KO and Ramos WT cells upon stimulation with PMA. Data presented is normalized to the respective unstimulated controls. (**d**) Western blots for Ramos WT and ADPGK KO cells at D0, 2 and 7 with β-Actin as loading control. (**e)**, (**f**) Quantification of band intensities using ImageJ software. All graphs are representative of three independent experiments (**g**) Homotypic aggregates observed at 5 × magnification under a light microscope at D7 in PMA stimulated WT and KO cells. WT, KO: mean of values from two wild-type and two *ADPGK* knock-out cell lines, obtained from three independent experiments. Scale bar corresponds to 400 µm. For western blots, TC is transfection control and the two knock-out lines as KO-1 and KO-2. *y-axis* in all graphs represents respective raw values normalized to unstimulated WT cells. (*p < 0.05; **p < 0.01, ***p < 0.001, calculated using Welch’s t-test for significance).

Furthermore, forward scatter (FSC) and side scatter (SSC) data representing cell size and granularity, respectively^26^ showed increase in FSC and SSC values in both cell lines post stimulation, showing a large increase in size and increased cytoplasm:nucleus content. However, on D7 there was a significant increase in side scatter for KOs compared to WT most likely depicting higher number of apoptotic cells in the stimulated KOs which failed to differentiate (Fig. 2c). The morphological changes linked with apoptosis in KO cells were confirmed by AnnexinV staining^31^ where KO cells showed almost 70% higher staining of the apoptotic marker compared to WT cells (Fig. 2a, b, e). Gating strategy for selection of lymphocytes was employed to exclude cell debris from the analysis and is depicted in Fig. 2d. The apoptotic profile of these cells was also assessed by analysing the content of cleaved Caspase-3^61^ at D0, D2 and D7 post stimulation (Fig. 2f). We noticed an increase in the content of cleaved Caspase-3 in ADPGK KO cells compared to Ramos WT at the differentiation phase (D7) and thus indicated cells which failed to proceed through activation induced differentiation, and succumbed to apoptosis. Moreover, activated B-cells proceeding through the terminal stages of differentiation are bound to have increased content of the ER stress marker *XBP1(s)* (spliced) transcript compared to the un-spliced *XBP1* mRNA^32,33^. Hence we analysed the levels of spliced/un-spliced *XBP1* in WT Ramos and ADPGK KO cells. We found the ratio of spliced to un-spliced *XBP1* to be higher in KO cells compared to WT in unstimulated state (almost two folds) (Fig. 3a), which is not a characteristic of Burkitt’s lymphoma. Previous studies have shown very low or absent transcript levels of *XBP1* in Burkitt’s lymphoma patient biopsies, typical of an undifferentiated lymphoma^34^. This signalled stressed cellular machinery in ADPGK KO cells even in unstimulated state. At day two post stimulation, we observed a decrease in *XBP1* splicing in both WT and KO cells, which could be explained by increased proliferative, glycolytic phenotype in the activation phase (Fig. 3a). Further, at seven days post stimulation with PMA, the trend reversed dramatically in favour of a differentiated state with almost six to eight-fold increase in *XBP1* spliced/un-spliced level in Ramos WT cells. The KO cells, though, exhibited a much lower comparative increase, with only two folds higher spliced mRNA levels of *XBP1* compared to its expression at two days post stimulation with PMA, again pointing towards impaired differentiation (Fig. 3a).

To ascertain if the Ramos WT cells have indeed stopped cycling at D7 and entered the differentiation phase, we performed RT-PCR expression analysis of the cell-cycle regulating gene *CDKN1A* (p21), which is also an important regulator of B-cell differentiation and lymphoid malignancies^62–64^. p21 expression was induced in activation phase (D2) of Ramos WT cells and maintained at slightly reduced levels in the differentiation phase (D7), signifying pre-plasmablasts (Fig. 3b). ADPGK KO cells on the other hand, exhibited significantly lower levels of p21 in both the activation and differentiation phase (Fig. 3b). Apoptosis observed in ADPGK KO cells, shown by Annexin V staining (Fig. 2a) could also be attributed to lack of p21, as high levels of p21 are known to prevent cells from apoptosis and assist in differentiation^64^. We additionally performed MTT (3-(4,5-dimethylthiazol-2-yl)-2,5-diphenyl tetrazolium bromide) assay to assess the proliferation of Ramos WT and ADPGK KO cells upon PMA induced activation and differentiation (Fig. 3c). Consistent with our hypothesis, MTT assay analysis showed high proliferation rate for Ramos WT cells in the activation phase (D2) which ceased at the onset of differentiation (D7). ADPGK KO cells displayed significantly reduced proliferation during the activation phase (D2), which went down further at D7, depicting apoptotic cells (Fig. 3c).

Recent studies point to a correlation between the levels of Protein Disulphide Isomerase (PDI) and the differentiated state of a cell^35–37^ as well as increased PDI expression upon cellular stress conditions exerting a pro-survival effect^38–40^. Differentiated B-cells secrete immunoglobulins, which are rich in disulphide bonds, and whose formation in the endoplasmic reticulum (ER) is catalysed by (PDI)^41^. With several studies pointing to this correlation, we quantified the protein content of PDI in Ramos WT and ADPGK KO unstimulated, D2 and D7 cells. The protein blots revealed a gradual increase in PDI levels in all cell lines upon activation, with levels increasing up to four-folds at D7 for WT cells (Fig. 3d, e). At all the measured time points, PDI protein levels in WT cells remained significantly higher than ADPGK KO cells (Fig. 3d, e). Overall, PDI was upregulated in accordance with the increased differentiation status of WT cells thus potentially favouring its more proliferative phenotype.

The increased ER content associated with activation of B-cells was measured via the widely known ER-marker Calreticulin. Protein blots showed that Calreticulin uniformly increased post PMA stimulation in both WT and ADPGK KO cells depicting that the failure to upregulate ER stress machinery was not linked to total ER-content in these cells (Fig. 3d, f).

Further, B-cells are also known to form homotypic aggregates upon stimulation by appropriate antigen^23,42^ and the interaction supports the clonal expansion and activation of lymphocytes^43^. We indeed found large aggregates of cells starting to form as early as 48 h post stimulation with PMA in Ramos WT cells, which increased several folds in size up to D7. KO cells, on the other hand, displayed significantly smaller homotypic aggregates providing a visible evidence of hindered differentiation in these cells (Fig. 3g).

### ADPGK KO B-cells are metabolically toxified

**Figure. 4:**
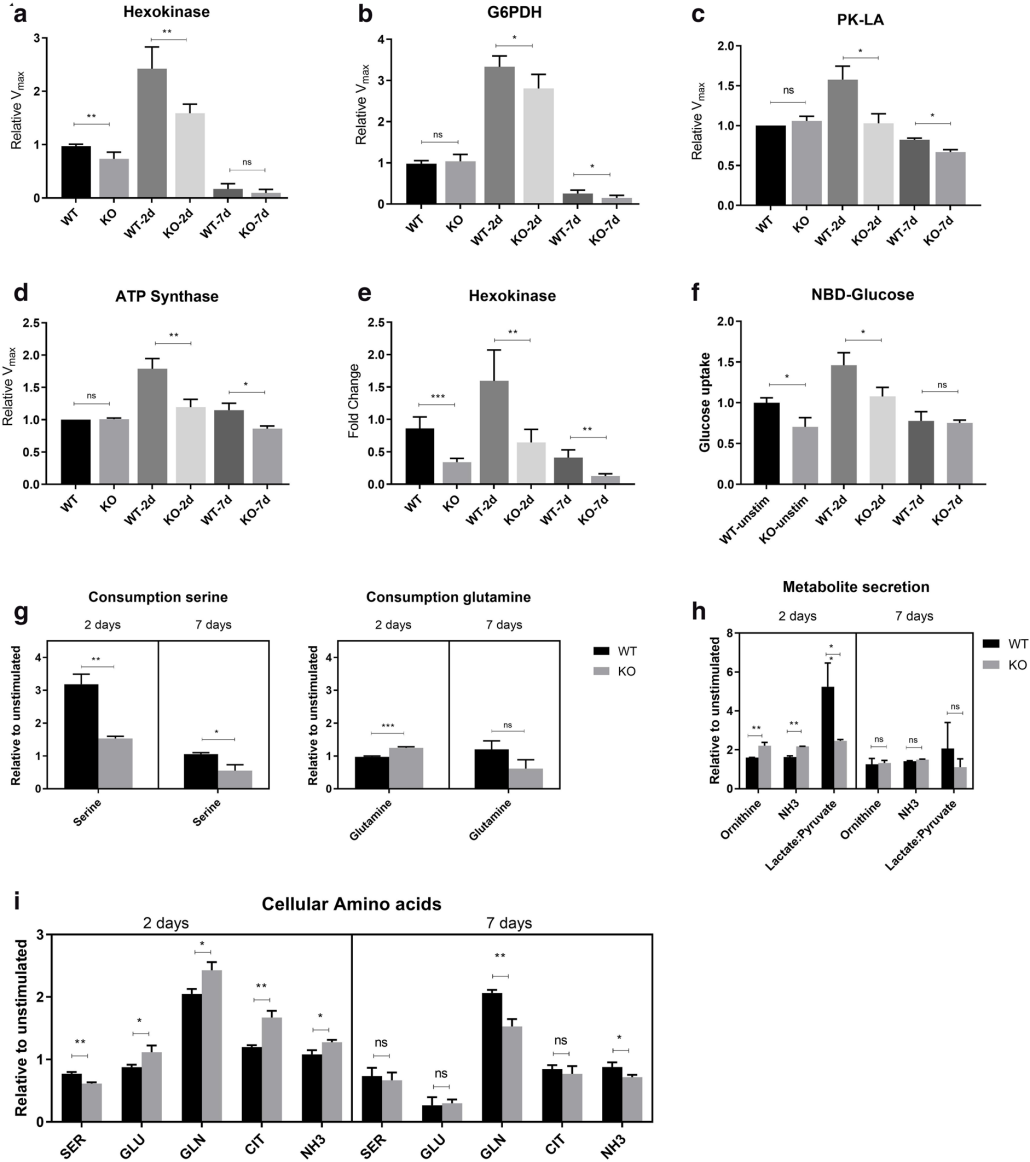
ADPGK KO B-cells are metabolically toxified. (a)*–*(d) ADPGK KO and WT cells were analysed for aerobic glycolysis upon stimulation with PMA by measurement of enzyme activities of Hexokinase-2, G6PDH (Glucose 6-phophate dehydrogenase), Pyruvate kinase M2-dimeric (PK-LA) and ATP synthase spectrophotometrically; RT-qPCR for hexokinase-2 (**e**) for gene expression, and (**f)** accumulated FITC-NBD-glucose (2-(*N*-(7-Nitrobenz-2-oxa-1,3-diazol-4-yl) Amino)-2-Deoxyglucose) via flow cytometry for glucose uptake. (**g**) Serine and Glutamine consumption in Ramos WT and ADPGK KO cells at D2, D7 normalized to their respective unstimulated values. (**h**) Secreted metabolites in culture media at D2, D7; lactate:pyruvate levels for estimation of Warburg phenotype and Ornithine, NH_3_ values for glutamine metabolism, normalized to respective unstimulated conditions. (**i**) Intracellular amino acids and metabolites measured in ADPGK KO and Ramos WT cell lysates at two and seven days post stimulation with PMA. Data shown are normalized to the respective unstimulated controls. SER- Serine, GLU- Glutamic acid, GLN- Glutamine, CIT- Citrate, NH_3_-Ammonia. All graphs are representative of three independent experiments. WT, KO: mean of values from two wild-type and two *ADPGK* knock-out cell lines, obtained from three independent experiments. *y-axis* in all graphs, except amino-acid consumption and metabolite secretion, represents respective raw values normalized to unstimulated WT cells. (*p < 0.05; **p < 0.01, ***p < 0.001, calculated using Welch’s t-test for significance).

Next, we aimed at measuring the hallmarks of Warburg effect in WT and ADPGK KO cells two days and seven days post stimulation with PMA. We measured glycolytic enzyme activities of Hexokinase-2 (HK2),Glucose 6-phosphate dehydrogenase (G6PDH), Pyruvate Kinase M2-dimeric (low affinity for phosphoenolpyruvate; PK-LA), and mitochondrial respiratory chain complex ATP Synthase (Complex V)^14,44^ in Ramos WT and ADPGK KO cells. WT cells stimulated with PMA showed significant increase, (1.5–3 folds) in glycolytic enzyme activities (Hexokinase-2 , G6PDH and PK-LA) and respiratory chain complex whereas the ADPGK KO cells displayed a significantly reduced activation-based increase in enzyme activities compared to WT cells post two days (Fig. 4a–d). At seven days, the activity values dropped to almost 20 percent of the unstimulated state activities in both cell type but with WT cells displaying a higher residual activity than KOs, indicating non-proliferating but viable cells (Fig. 4a–d). Gene expression values measured via RT-qPCR for Hexokinase-2 followed the same pattern as enzymatic activities and suggested a stably reduced glycolytic phenotype of KO cells (Fig. 4e).

Next, we measured NBD-glucose (2-(*N*-(7-Nitrobenz-2-oxa-1,3-diazol-4-yl)Amino)-2-Deoxyglucose)^45^ uptake in two days and seven days-PMA stimulated WT and KO cells. As shown in Fig. 4f, in the 48 h stimulated cells, we observed an increased uptake in all cells with respect to unstimulated cells but WT cells exhibiting significantly higher amounts of NBD glucose content compared to KO’s (Fig. 4f). At D7, despite no significant difference in glucose uptake between WT and KO, there was a strong reduction in uptake rate compared to D2 in all cells (Fig. 4f) understating a ceased activation phase and differentiated or apoptotic cells originating from Ramos WT or ADPGK KO respectively.

After detection of low glycolytic profile of ADPGK KO cells, we next wanted to investigate the amino acid uptake rate of these cells as replenishment for hampered glucose processing. The results, as depicted in Fig. 4g show significantly higher consumption of serine by Ramos WT cells, compared to ADPGK KOs, at 2 days post PMA stimulation. The higher increase in consumption of glutamine in stimulated state compared to unstimulated state (ratio, two days stimulated:unstimulated) in ADPGK KO cells was noteworthy (Fig. 4g). High dependence on glutamine as metabolite was further reflected by dramatic increase in free ammonia (NH_3_) levels in media of ADPGK KO cells in unstimulated and stimulated states, which reflected the consumption of glutamine (Fig. 4g). Ornithine, which is originally not present in media formulation used for these cells, and was probably a by-product of glutamine metabolism, was also detected at values correlating with NH_3_ levels and underlining glutamine dependent energy generation and biosynthesis by ADPGK KO cells (Fig. 4g, h).

Lactate and pyruvate content in culture media obtained from two days and seven days stimulated cells was analysed as a quantitative estimate of aerobic glycolysis. The levels of lactate:pyruvate rose in all cells at 2 days post stimulation by at least two folds, signifying actively proliferating cells (Fig. 4h). However, the comparative data again hinted towards a much-reduced aerobic glycolysis occurring in ADPGK KO cells with the levels of lactate:pyruvate in 2 days stimulated cells almost half of that in Ramos WT cells (Fig. 4h).

We also analysed the levels of cellular amino acids and metabolites (Ser, Glu, Gln, Cit and NH_3_) in cell lysates from Ramos WT and ADPGK KO cells at D0, D2 and D7. The data, depicted in Fig. 4i, correlates with the amino acid consumption pattern of these cells and again shows that ADPGK KO cells upregulate their glutamine dependent energy synthesis to bypass the dysfunctional glycolysis in these cells^14^.

### *MYC* expression and mutational status are dependent on glucose metabolism

**Figure. 5:**
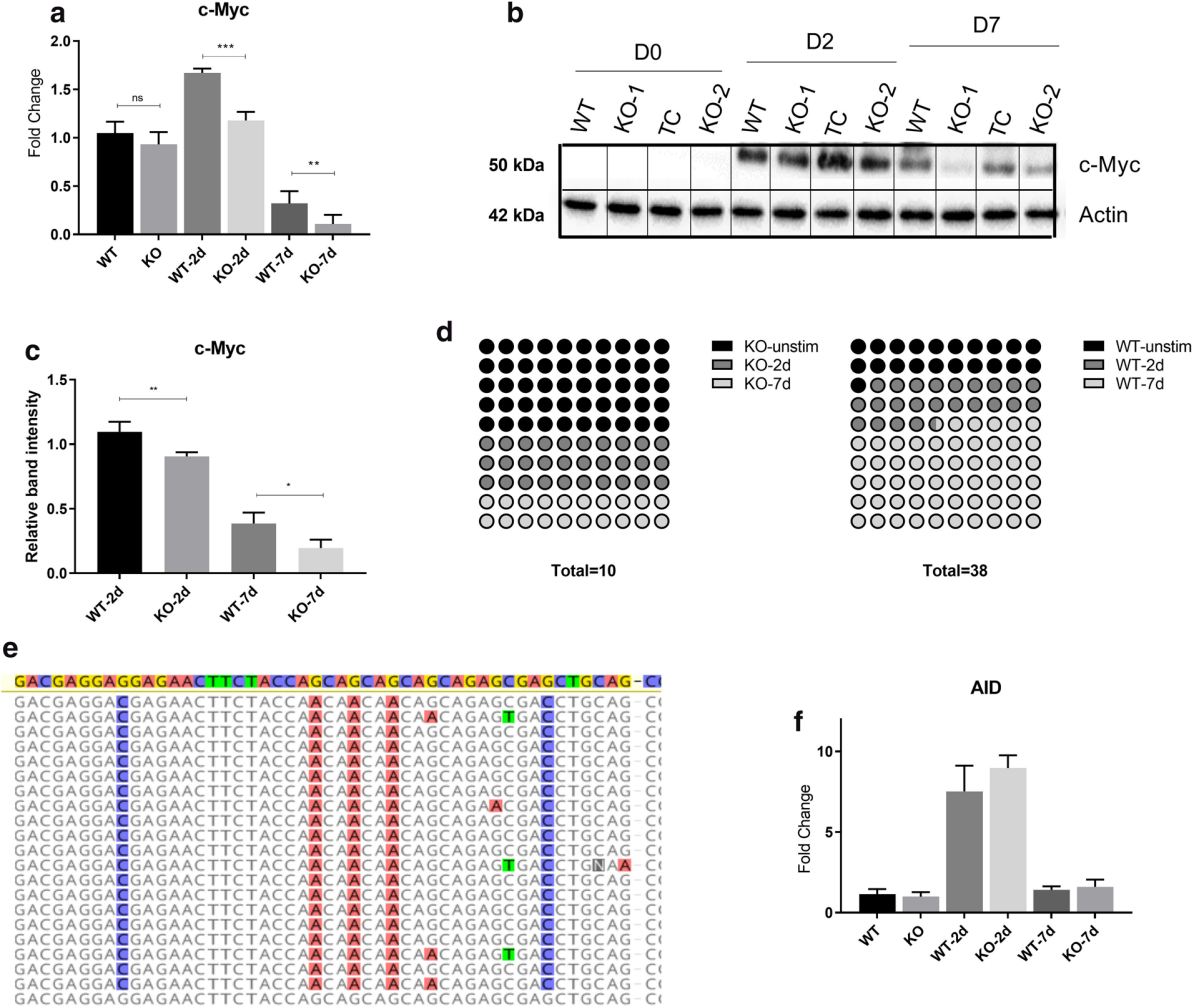
*MYC* expression and mutational status are dependent on glucose metabolism. *MYC* (translocated and wild-type) expression in ADPGK KO and WT cells at different time points post stimulation was measured via RT-qPCR and protein content via western blots. (**a**) Expression of *MYC* at D0, D2 and D7 post stimulation. *y-axis* represents fold change normalized to unstimulated WT cells. (**b**) Protein blot of MYC and β-Actin loading control, with quantification of bands in (**c**) using ImageJ software. (**d**) Transcripts obtained at D0, D2 and D7 were sequenced and aligned with wild type *MYC* to analyse mutations in translocated allele. Panel **d** is a 10 × 10 dot plot for representation of observed mutations in WT and KO cells at D0, D2 and D7 post stimulation. Each dot corresponds to one percent. Total number of observed mutations for each cell line are given below the plots. (**e**) Representative figure showing the distribution of random mutations and their preferential targeting to AGC sites with respect to wild type *MYC* (topmost highlighted sequence). (**f**) RT-qPCR expression data for AID (activation-induced cytidine deaminase) in WT and KO cells at different time points post stimulation. Expression analysis and protein blots are representative of three individual experiments. Error bars show + /- s.e.m. Mutational analysis was performed with sixty-five individual *MYC* transcripts obtained from WT and KO cells at different time points. Sequences were aligned in Geneious software and figures exported are depicted. WT, KO: mean of values from two wild-type and two *ADPGK* knock-out cell lines, obtained from three independent experiments. (*p < 0.05; **p < 0.01, ***p < 0.001, calculated using Welch’s t-test for significance).

Myc proto-oncogene, known to be the major regulator and driver of Burkitt’s lymphoma, we wanted to assess the effect of PMA stimulation on expression of *MYC* (both wild type and translocated). RT-qPCR results showed a significant increase in *MYC* transcript levels in Ramos WT cells at two days post activation (Fig. 5a), signifying metabolically active proliferating cells. ADPGK KO cells however displayed a reduced upregulation of *MYC* expression at D2 compared to Ramos WT cells (Fig. 5a). At day seven post stimulation, we observed a reduction in *MYC* transcript levels, to almost 30 percent of base level values in Ramos WT cells but to nearly 10 percent of base level in ADPGK KO cells (Fig. 5a). These results were confirmed by protein blots for MYC protein as well (Fig. 5b, c); however, we failed to detect MYC in unstimulated state in all samples via western blot suggesting selective expression of translocated *MYC* in these cells.

Next, to test the mutational status of the translocated transcripts being produced at different time points post PMA stimulation, we sequenced the *MYC* transcripts from Ramos WT and ADPGK KO cells at different stimulation points (two-days and seven-days) and aligned them with known sequences of translocated and wild-type *MYC* in Ramos Burkitt’s Lymphoma cells. The results depicted as per expectations, a high number of mutations accumulated in *MYC* transcript in Ramos WT cells and ADPGK KO cells (Fig. 5e). Several of the mutations were conserved throughout the analysed sequences. Most of the sequences matched with the known mutations in translocated *MYC* allele of Ramos BL cells and patient biopsy samples of Burkitt’s lymphoma^8,9^. However, some of the conserved mutations were novel and depicted additional effect of hypermutation during culture of these cells (Fig. 5e)^9^. Insertions and deletions of several bases were observed along with point mutations (Fig. 5e). Upon sequencing of two day and seven days stimulated Ramos WT and ADPGK KO cells, we obtained wild type *MYC* sequence in almost 50% of the cases. This finding is in first sight, in contradiction to previous studies which reported that only the translocated *MYC* allele is expressed in Ramos BL cells^9^. However, the sequences used in previous studies were only obtained from unstimulated cells and true to that, we obtained only translocated *MYC* allele from non-PMA stimulated cells.

Next, we were interested in random non-conserved point mutations which had likely accumulated due to culturing and PMA based activation of Ramos WT and ADPGK KO cell lines. Analysis of almost 60 WT and KO sequences revealed 38 independent mutations in WT cells and just 10 mutations in KO cells (Fig. 5d). Moreover, out of the 38 point mutations in WT cells, 30 were found in PMA stimulated conditions (two day + seven day). More than 2/3^rd^ of these (21 precisely) were found in seven day stimulated cells, clearly pointing to a role of activation induced hypermutation in the observation (Fig. 5d). On the other hand, ADPGK KO cells displayed half (5/10) mutations in the stimulated state, out of which just two were in seven days stimulated cells (Fig. 5d). Knowing that ‘AGC triplets’ are a hotspot for activation induced cytidine deaminase (AID) targeted mutations in activated GC B-cells^9,46^, we analysed the location of mutations with respect to AGC (or GCT, reverse complement) sites. Indeed, 20/38 mutations were at AGC triplets, out of which 18 were found in sequences from stimulated Ramos WT cells (Fig. 5d). A random mutational targeting with respect to the consensus translocated *MYC* sequence would have yielded maximum 13% hits on AGC triplets. Also, 36/38 targets were at G/C sites. There was also a preference for transitions over transversions with 50% observed mutations in WT cells occurring as transitions (randomly expected 33%). Such highly specific and preferential mutational targeting is a characteristic of immunoglobulin hypermutation^9,11^ and clearly reflected a much higher degree of organized mutations in ADPGK WT cells compared to the KOs. The differences between Ramos WT and ADPGK KO sequences are summarized in Table 3 of the Supplementary information.

To determine the underlying reason for increased accumulated mutations in WT cells, we measured Activation-induced Cytidine Deaminase (AID) expression in ADPGK KO and Ramos WT cells. AID expression, analysed at different time points post stimulation in KO and WT cells was not significantly different, suggesting an underlying role of transcriptional activity in the accumulation of mutations in WT cells (Fig. 5f).

### ADPGK KO leads to reduced migration of macrophages to tumour

**Figure. 6:**
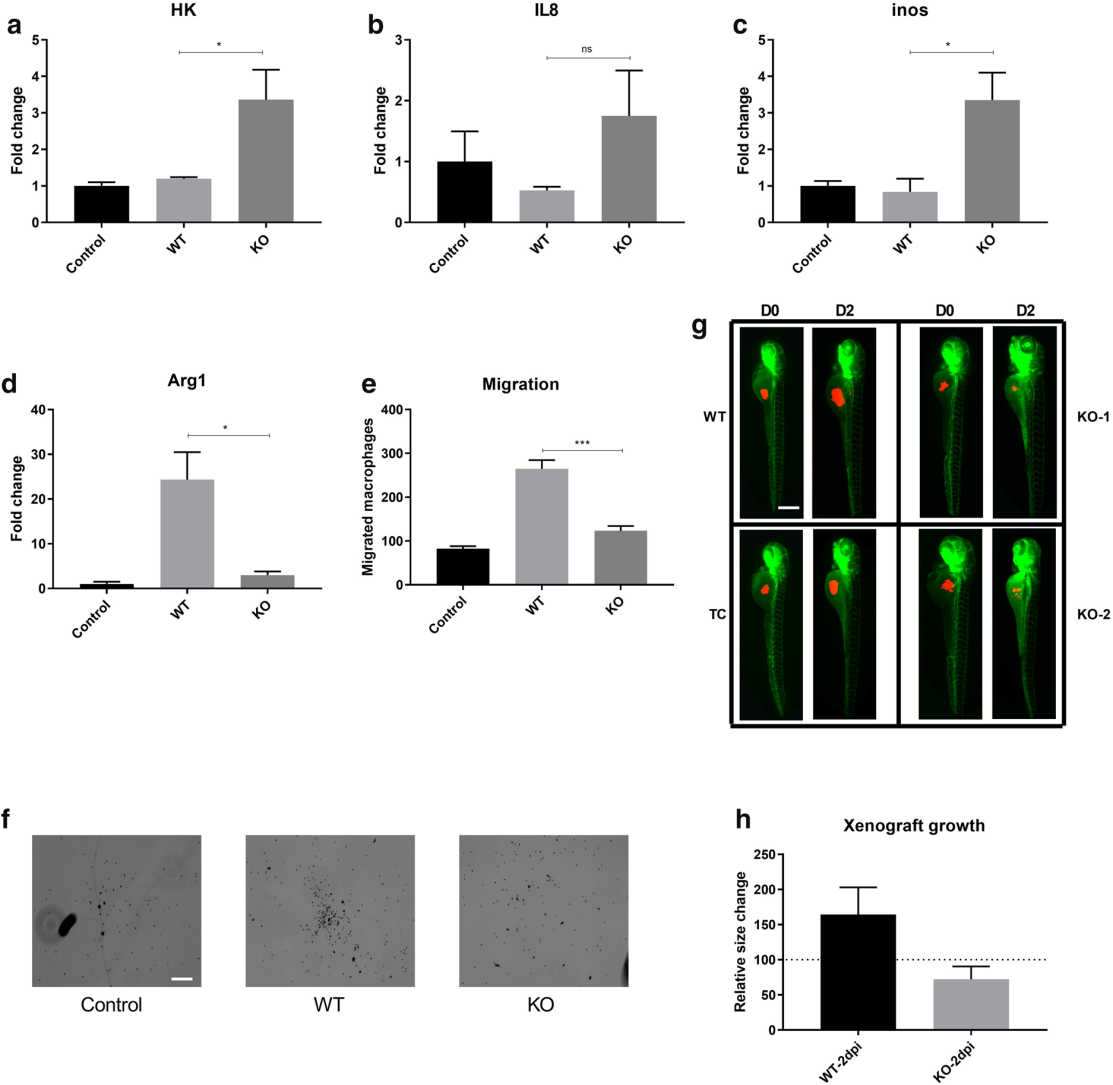
*In-vitro* and *in-vivo* analysis of tumour aggressiveness in ADPGK KO and Ramos WT cells. (**a**)**–**(**d**) PMA activated THP-1 monocytes were co-cultured with stimulated WT Ramos or ADPGK KO cells for a period of 48 h. Monocyte-macrophages collected post co-culture with Ramos WT or ADPGK KO cells were analysed via RT-qPCR for expression of M1, M2 markers in form of Hexokinase, IL-8, inducible nitric oxide synthase (iNOS) and Arginase-1. PMA activated THP-1 cells without a co-culture setup served as control for the experiment. (**e**) The migration of THP-1 monocyte/macrophages was studied in presence of WT or ADPGK KO cells. Media without WT/KO cells served as control. The quantification of migrated THP-1 cells post 6 h of incubation is represented as cell numbers on *y-axis*. (**f**) Microscopic images representing migrated THP-1 cells upon co-culture with WT and KO cells (**g**)***, ***(**h**) Approximately 200–250 CM-diI labelled cells (WT or KO) were injected in yolk of 48 h post fertilization (hpf) *kdrl:GFP* zebrafish larvae. Images in (**g**) show the progression of xenografted cells over two days post injection (D0–D2) in Ramos wild type (WT) and transfection control (TC) and ADPGK knock-out (KO-1, KO-2) cells. Xenograft area quantified using ImageJ for 50 larvae injected with Ramos WT and another 50 with ADPGK KO cells and mean calculated over three individual experiments is shown in (**h**). WT, KO: mean of values from two wild-type and two *ADPGK* knock-out cell lines in all bar-graphs. Error bars show + /− s.e.m. Scale bar corresponds to 400 µm. Dpi is Days post injection. (*p < 0.05; **p < 0.01, ***p < 0.001, calculated using Welch’s t-test for significance).

Immune cells, especially macrophages, migration to Burkitt’s lymphoma microenvironment is often considered as one of the most important factors contributing to aggressiveness of the disease^47,48^.The macrophages which are chemotactically or otherwise attracted towards the tumour niche, the Tumour Associated Macrophages (TAMs), are polarised to and serve as tumour growth promoting, anti-inflammatory, M2 macrophages^49^. To study the effect of ADPGK KO on regulation of immune cell chemotaxis, the migration of THP-1 monocyte/macrophages was studied in presence of Ramos WT or ADPGK KO cells. The quantification of migrated THP-1 cells post 6 h of incubation showed a marked difference between Ramos WT and ADPGK KOs, with the latter displaying a reduced number of THP-1 cells which underwent migration, similar to the negative control (only media, no cells) (Fig. 6e, f). Ramos WT exhibited migration rates more than double compared to its KO counterparts, indicating a significant loss in chemotactic attraction capabilities of Burkitt’s lymphoma cells upon removal of *ADPGK* (Fig. 6e).

### Macrophage M1 polarisation increases with KO of *ADPGK*

Subsequently, we aimed at replicating the tumour-immune cell interaction at an in vitro level to understand the phenotype bestowed upon the macrophages by lymphoma cells. PMA activated THP-1 monocytes were co-cultured with stimulated Ramos WT or ADPGK KO cells for a period of 48 h, which corresponds to the activation state of B-cells signifying high proliferation and high glycolytic activity found in aggressively growing lymphomas. Monocyte-macrophages collected post co-culture displayed a greater shift towards the M2 polarised state when incubated with Ramos WT cells compared to ADPGK KOs, which was evident by the reduced expression of glycolytic enzymes such as hexokinase (HK), M1 marker Inducible nitric oxide synthase (iNOS1) and pro-inflammatory cytokine IL8, and increased expression of M2 marker Arginase 1 (Arg1) (Fig. 6a–d)^49–52^. Macrophages collected from ADPGK KO cells co-culture on the other hand displayed enhanced expression of HK, which was significantly increased compared to the cells stimulated with PMA alone and not incubated in a co-culture setup, depicting a shift towards high glycolytic, M1 polarised state (Fig. 6a). Expression of other M1 markers such as IL8 and iNOS were also upregulated in cells collected from ADPGK KO co-culture (Fig. 6b, c). On the other hand, the expression of M2 marker, Arg1, was reduced in macrophages obtained from ADPGK KO co-culture (Fig. 6d). Overall, the expression data for M1/M2 markers clearly showed that only macrophages co-cultured with Ramos WT cells were increasingly polarised to a tumour-assisting M2 phenotype.

### ADPGK KO affects the growth of lymphoma xenografts in Zebrafish larvae

To monitor the development of lymphoma xenografts in zebrafish larvae over a period of two days, we injected PMA activated Ramos WT and ADPGK KO cells in the yolk of *kdrl:GFP* zebrafish larvae 48 hours post fertilization (hpf). Images were taken over 48 h (D0 and D2) and the growth of tumour was quantified. Images taken immediately after injection (D0), showed a near equal distribution of Ramos WT and ADPGK KO xenografted cells in zebrafish yolk (Fig. 6g). Post 48 h (D2), we observed a significant increase (up to 50% increase than D0) in the size of xenografted mass in 43/50 zebrafish larvae in case of Ramos WT cells (Fig. 6g, h). Larvae injected with ADPGK KO cells showed growth of the xenografted mass only in 16/50 injections, plus the growth was significantly hampered compared to their WT counterparts (Fig. 6g, h). In the remaining injected zebrafish, the xenografted mass shrinked in most of the larvae (21/34) while being consistent in size in others, likely reflecting the inability of ADPGK KO cells to grow in an external environment.

## Discussion

In this study we aimed at analysing the effect of ADPGK on regulation of aerobic glycolysis in Ramos BL cells, in terms of their malignant and differentiated status upon activation. We found reduced tumour aggressiveness upon activation, both in vitro and in vivo zebrafish, and a hindered activation mediated differentiation of ADPGK KO cells. These findings were underlined by reduced *MYC* transcript and protein levels as well as diminished accumulation of activation induced mutations in the translocated *MYC* allele in ADPGK KO cells. These effects were further amplified upon mitogen activation with PMA mainly at D2 and correlated with the increased ADPGK expression and activity two days post PMA stimulation. Cells lacking a glucose sensor and aerobic glycolysis regulator in form of ADPGK failed to garner sufficient resources and energy during the activation phase to proceed though the metabolically demanding process of cellular differentiation and succumbed to apoptosis.

A reduced Warburg phenotype upon activation for ADPGK KO cells was reflected by study of glycolytic enzyme activities (HK2, G6PDH, PK-LA and ATP Synthase) and expression (*HK2*), and pointed to a direct link between ADPGK and BL cell metabolism. Detection of fluorescently labelled glucose, displayed a reduced glucose uptake by ADPGK KO cells, reflecting the metabolic changes seen through enzymatic measurements. The observed reduction in aerobic glycolysis in ADPGK KO cells was further confirmed via measurement of amino acids (serine, glutamine and ornithine) and lactate/pyruvate levels. Overall, the data suggested that hampered induction of Warburg effect underlines the failure of ADPGK KO cells to fully proliferate and differentiate upon PMA activation.

We observed a perturbation of differentiation markers in ADPGK KO and WT cells. Markers of differentiation in form of CD20, CD138, *XBP1*, increase in cell size and granularity, and homotypic aggregation provided a clear view of well differentiated WT cells and a delayed entry to differentiation of ADPGK KO cells. Ultimately ADPGK KO cells ended with apoptosis, as observed from SSC, AnnexinV staining and cleaved Caspase-3 content in these cells. We also observed dysregulation of an important cell-cycle regulator and controller of differentiation, *CDKN1A*, in ADPGK KO cells. MTT assay further fortified our hypothesis and displayed a much reduced proliferative capacity of KO cells upon PMA stimulation. Overall, ADPGK KO cells were unable to cope up with the metabolic stress of activation induced differentiation and succumbed to apoptosis.

The non-negative values observed for B-lymphocyte antigen CD20 and overall low levels of CD138 plasma cell marker in Ramos WT cells proceeding towards differentiation signified a population not characteristic of plasma cells but probably a plasmablast transition state as also discussed in several other studies^26,27,29^. Pre-plasmablasts are known to secrete lower levels of immunoglobulins than plasma cells and do not display the standard plasma cell markers. Nonetheless, the higher residual expression of CD20 in ADPGK KO cells is important in the light of using chemotherapeutics such as Rituximab^53^ which depend on binding to CD20 and might also prove useful in lymphomas exhibiting resistance to this drug via shedding off the drug-CD20 complex. Analysis of ER stress regulators such as *XBP-1* and PDI further depicted a marked difference in response to increased unfolded protein content in the ER between Ramos WT and ADPGK KO cells. During the process of differentiation, ADPGK KO cells hampered in their ability to cope up with the highly increased flux of proteins (Ig’s) associated with B-cell activation and differentiation. Additionally, lower content of PDI in ADPGK KO cells pointed towards a reduced proliferative phenotype of these cells as the protein exhibits a pro-survival/anti-apoptotic effect in cancer cells^39,40^.

We found wild type *MYC* expression increasing upon activation and going down upon differentiation to stop cellular proliferation, as also known from B-cell differentiation models^54–57^. The reduction of overall levels of MYC at day seven signified a differentiated status of these cells^57,58^. In case of Burkitt’s lymphoma, translocated *MYC* becomes a target of somatic hypermutation (SHM) and activation induced cytidine deaminase (AID) mediated mutational activity^9,59^. However, accumulation of mutations, besides depending on AID activity, more importantly depend on the transcriptional rate of the target locus with a highly transcribing locus accumulating proportionally higher number of mutations^60^. With observed AID expression levels nearly the same in Ramos WT and ADPGK KO cells,it is evident that a higher transcriptional activity of translocated *MYC* under the IgH promoter was responsible for the intriguingly large difference in accumulated mutations between WT and ADPGK KO cells, thereby showing the increased proliferative, activated phenotype of WT cells upon stimulation.

The findings were further cemented by in vitro measures of tumour aggressiveness via migration and co-culture, showing a reduced capability of ADPGK KO cells to transform tissue resident macrophages for their advantage. The process of macrophage transformation is considered as a crucial step in growth and immune evasion of tumours and curbing this effect could help curb malignancies (esp. lymphomas) heavily relying on this mechanism. Extended to in vivo tumour growth quantification in zebrafish larvae, the ADPGK KO cells again displayed a reduced malignant status as evident from their failure to grow in a natural habitat, thus providing a definitive cue to the importance of ADPGK in regulating aerobic glycolysis mediated tumour aggressiveness.

The role of ADPGK in regulation of Warburg effect now being extended to B-cell malignancies after the recent study^18^ on its role in T-cell activation and aerobic glycolysis, a potential limitation of the study at this point could be that more representative cell lines and patient samples might be needed to generalize and fortify the role of ADPGK in other malignancies.

Differentiation and tumour aggressiveness, though complementary, but both depending on aerobic glycolysis for at least a part of their cycle, we have thus uncovered a novel target in the form of ADPGK on the cross-roads of these pathways, and its targeting via development of novel therapeutics could prove to be indispensable for curbing aggressively growing malignancies.

## Methods

### Cell culture

Ramos (RA-1, ATCC CRL-1596) B-lymphocytes and THP-1 (ATCC TIB-202) monocytes were maintained as a suspension culture in RPMI-1640 media supplemented with GlutaMax (Thermo Fisher Scientific), 10% fetal bovine serum (FBS) and 1% Penicillin/Streptomycin. Cell cultures were incubated for optimum growth at 37° C and 5% CO_2_ in a humidified atmosphere and fresh media was added every 2–3 days. Stimulation of cells was induced by PMA (phorbol 12-myristate 13-acetate) at 10 ng/ml for THP-1 cells and at 50 ng/ml for Ramos BL cells.

### *ADPGK* knock-out

*ADPGK* knock-out (KO) Ramos cells were generated using the CRISPR/Cas9 technology. Exon-2 in *ADPGK* gene was targeted, which also codes for the glucose binding site of the translated protein, thereby providing catalytic function. CRISPR/Cas9 plasmid expressed guide-RNA designed against exon-2 under a U6 promoter and a green fluorescent protein (GFP) under the cytomegalovirus (CMV) promoter. Target site was GTCAATGCATGTGTTGATGTGG in exon 2. We used un-transfected and GFP-plasmid transfected cells as positive control for ADPGK and as Transfection Control (TC) respectively for subsequent experiments. All transfections were performed using Bio-Rad Gene Pulsar Electroporation system at recommended conditions for lymphocytes. Transfected cells were sorted using FACS for GFP into 96 well plates. Sorted single cells were allowed to grow in standard cell culture conditions until the visible formation of colonies. Clones were selected by detection of 46 kDa ADPGK band in protein lysates obtained from the colonies by Western blots using anti-ADPGK antibody (Sigma-Aldrich, HPA045194) and additionally by DNA sequencing. For sequencing, DNA was obtained from clones using DNeasy Blood and Tissue Kit (Qiagen) following the manufacturer’s protocol and PCR (polymerase chain reaction) amplified using the Q5 High-Fidelity DNA polymerase (New England Biolabs). The PCR products were sent for sequencing to Microsynth SEQLAB (Göttingen, Germany). Sequencing results were analysed via BLAST (Basic local alignment search tool, NCBI) against *ADPGK* wild-type sequence.

### Immunoblot analysis

Sodium dodecyl sulfate polyacrylamide gel electrophoresis (SDS PAGE) was used for separating the proteins in cell lysates. Cell lysis was performed with RIPA buffer using a pellet of 5 million cells suspended in the buffer and subjected to 10 × 5 s cycles of sonication. Supernatant containing the extracted proteins was resolved by SDS PAGE in 10% polyacrylamide gels. Equal volume for all samples adjusted to 40 µg protein content were loaded in the gels. The separated proteins were immunoblotted on PVDF (polyvinylidene fluoride) membranes according to standard procedure and blocked in TBST with 5% non-fat dry milk. Membranes were incubated overnight in primary antibody in blocking buffer and analysed by HRP-conjugated secondary antibody next day using chemiluminescent substrate (ECL). Phusion GelDoc system was used for visualization of results and quantification of band intensity was performed with ImageJ software (NIH, https://rsbweb.nih.gov/ij/). The list of primary and secondary antibodies is mentioned in Supplementary information.

### Quantitative RT PCR analysis

Total RNA was extracted from cells using TRIzol reagent (Thermo Fisher Scientific) using supplier’s protocol. Approximately 5 million cells were used per extraction. Isolated RNA was subjected to DNase treatment (DNase1, Recombinant, Sigma-Aldrich) to remove contaminating DNA. 1 µg of resulting purified RNA was used to prepare cDNA with the help of Maxima cDNA synthesis kit (Thermo Scientific). For expression analysis by RT-qPCR, we used SensiMix SYBR Hi-ROX Kit (Bioline) with template cDNA and primer concentration according to the manufacturer’s protocol. Primer sequences are provided in Supplementary data. All primers were previously verified for specificity by gel electrophoresis. PCRs were run on Bio-Rad CFX Connect RT-PCR Detection system at a preset melting cycle with annealing temperature specific for primer set. For quantification, 18S ribosomal RNA expression was used as an endogenous reference. Expression data was quantified using 2^−ΔΔCT^ method and stated as fold change in gene expression for each individual gene.

### Flow cytometry analysis

Ramos wild type and ADPGK KO, stimulated (2 and 7 days) and unstimulated, cells were stained with CD20 and CD138 fluorochrome conjugated antibodies (BD Biosciences) according to the manufacturer’s protocol. Cells from each study sample were also stained with FITC-AnnexinV and Propidium Iodide (BD Biosciences), for analysis of apoptosis. Flow cytometry measurements were performed on BD FACS Verse cytometer using BD FACS Suite application software. At least 100,000 cells were analysed for each sample and appropriate gating was applied to the populations to exclude cell debris and doublets. Data generated from positive gating was analysed using FlowJo software (Tree Star, USA). Flow cytometry data analysis was performed after gating on live B-cells using a pre-set analysis algorithm in FlowJo flow cytometry analysis software. Bound antigens for each sample were expressed as Relative Fluorescence Intensity (RFI), which is the ratio of the Median fluorescence intensity (MFI) of cells labelled with a specific antibody to that of unlabelled cells.

### MTT assay

Ramos WT and ADPGK KO cells were plated at a density of 10,000 cells/well of a 96-well plate, stimulated with PMA and cultured for up to seven days. At the end of stimulation time-points (D0, D2 and D7), cells were incubated at 37 °C with MTT (10 µl of 5 mg/ml per well of MTT solution, Sigma Aldrich, USA) for a period of 4 h. Solubilisation solution was added and the plate was shaken for 15 min to dissolve the formazan crystals. Absorbance was recorded at 570 nm and relative proliferation for each sample was calculated by dividing the absorbance value by the absorbance of control (D0) samples and multiplying by 100.

### Glucose uptake assay

Uptake of 2-NBDG (2-(*N*-(7-Nitrobenz-2-oxa-1,3-diazol-4-yl)Amino)-2-Deoxyglucose) glucose analogue was measured by flow-cytometry using “Glucose uptake cell based assay kit” (Cayman chemicals, Europe) according to the manufacturer’s protocol. Briefly, 5 million ADPGK KO and WT cells were seeded in 6 well plates and further stimulated with PMA for up to seven days. Analysis was performed with unstimulated, 2-day and 7-day stimulated cells (D0, D2 and D7). Cells were incubated in glucose free media for 4 h before analysis. At the end of glucose free incubation, 2-NBDG was provided at recommended concentration for 10 min and uptake measured via FITC measurement on flow cytometer. Cells incubated with Apigenin served as negative control for the experiment.

### Enzyme kinetics

For analysis of glycolytic enzyme activities, Ramos WT and KO cells (unstimulated, 2 and 7 days PMA stimulated) were lysed in Respiratory Chain Buffer (RCB). Around 15 million cells per sample were washed once with cold PBS (4 °C) post harvesting and dissolved in 700 µl cold RCB buffer. Samples were passed through 1 ml syringes fitted with 27G needles at least thirty times. Lysate was centrifuged at 300 g for 10 min to remove the debris and then at 3,000 g in a table top centrifuge to separate the mitochondrial pellet. The supernatant was spun at 9,500 g for 20 min to separate the Endoplasmic Reticulum (ER) pellet from the cytoplasmic supernatant. Supernatant, containing the glycolytic enzymes was used for analysis of Hexokinase-2 (isoform II) and Glucose-6-phosphate dehydrogenase (G6PDH) enzyme activities. The pellet was used for analysis of ADPGK enzyme activity. Enzyme activities were measured using colorimetric assays on a SpectraMax Plus 384 microplate reader (Molecular Devices) using SoftMax Pro Data Acquisition software in a 96-well plate. The enzyme activities were recorded as difference in absorbance at 340 nm to the absorbance at 400 nm and normalized to total protein content of the particular sample. ADP-dependent glucokinase (ADPGK) enzyme activity was measured as reduction of NADP in sample buffer containing 0.5 mM NADP, 1 mM glucose, 1 mM ADP and 0.05 U/ml glucose 6-phosphate dehydrogenase at pH 6.2 and 42 °C. Hexokinase activity was detected as NADP reduction in sample buffer containing 1 mM glucose, 1 mM ATP, 0.5 mM NADP and 0.05 U/ml glucose 6-phosphate dehydrogenase at 37 °C. For measurement of G6PDH activity, we measured the NADP reduction in sample buffer containing 0.5 mM NADP and 5 mM glucose 6-phosphate (G6P) at 37 °C. Pyruvate kinase M2-dimeric (PK-LA) was quantified by NADH oxidation in sample buffer containing 10 µM phosphoenolpyruvate, 1 mM ADP, 0.5 mM NADH and 2 U/ml LDH. Mitochondrial ATP synthase (Complex V) activity was measured in a sample buffer containing 250 μM NADH, 1 mM PEP, 2,5 U/ml LDH, 2 U/ml pyruvate kinase, 2 mM and 1 μM DQA (2-decyl-4-quinazolinyl amine, complex I inhibitor).

### Amino-acid analysis

Ramos wild type and ADPGK KO cells were seeded at 1 million cells/ml in 6-well plates and stimulated with PMA for two days and seven days, at the end of which, cells were harvested, and media was collected in separate 15 ml falcons. Media was further centrifuged at high speed and additionally passed through 0.35 µm filters to remove residual cells. 200 µl of the sample was mixed with 50 µl Sulfosalicylic acid and centrifuged at maximum speed on a table-top centrifuge. Supernatant was collected, and amino acid analysis was performed by injecting the samples into a Biochrom 30 + Amino acid analyser (Biochrom, UK) based on ion exchange chromatography, with resulting data expressed as µmol/litre. For analysis of cellular amino acids, cells were lysed using RIPA buffer and centrifuged at 13,000 g on a table top centrifuge to remove the cell debris. Supernatant was collected and analysed for amino acids as described above.

### Lactate/pyruvate measurements

Media obtained from cells, as described in amino-acid analysis, was analysed for lactate and pyruvate content. For pyruvate measurement the samples were mixed with pyruvate reagent 1 containing NADH, 1.5 M Tris Base and 0.2% HClO_4_ and added to reagent 2 containing LDH (lactate dehydrogenase). For lactate measurement, the samples were mixed with NADH, 0.6 M Glycine buffer and LDH. The completed samples were fed into and measured on a Beckman Coulter AU480 system (Beckman Coulter, USA) with final values obtained as mmol/litre.

### Mutational analysis of translocated *MYC*

cDNA obtained from unstimulated, 2-day and 7-day PMA stimulated ADPGK KO and WT cells was used to obtain *MYC* region coding for 3′ segment of exon 1 and 5′ plus middle segment of exon 2 via PCR using Q5 DNA polymerase. Analysis of this coding region would provide a direct cue to action of MYC as a transcriptional regulator. The product was purified using agarose gel electrophoresis and gel elution and cloned into TOPO-TA zero blunt vector (Thermo Fisher) as per kit guidelines. The plasmid was transformed into NEB-5 alpha competent *E.coli* (New England Biolabs) and plated for colony formation in LB-plates. 65 colonies from WT and ADPGK KO plate colonies were picked and directly sent for sequencing using “Ecoli NightSeq service” (Microsynth Seqlab). The obtained sequences were aligned with wild-type *MYC* and analysed for mutations using Geneious software (Biomatters Ltd., New Zealand).

### Macrophage migration assay

Migration assays were performed with 24-well Transwell inserts of 5 µm pore size, (Costar, Corning Incorporated, USA). THP-1 macrophages were seeded in Transwell inserts at 50,000 cells in 200 µl culture medium and stimulated by PMA for 24 h. Simultaneously, bottom chambers of 24 well cell-culture plates were seeded with 500,000 wild-type and ADPGK KO Ramos cells in 500 µl culture medium and stimulated with PMA for 24 h. The THP-1 macrophages were allowed to migrate for 6 h. After the completion of migration, Transwell inserts were washed with PBS (phosphate buffered saline) and the non-migrated cells present on the upper side of the inserts were removed with a cotton swab. The migrated cells on the lower side of the transwells were fixed overnight with 4% paraformaldehyde (PFA) at 4 °C and then stained with DAPI (4′,6-diamidino-2-phenylindole) (1:10,000). The DAPI-stained cells were imaged using Fluorescent microscopy (Leica DMI 4000B, Leica Microsystems) and later counted using the ImageJ software. Inserts incubated with media (no lymphocytes) were used as control.

### Macrophage-Ramos co-culture

To reproduce the tumour-macrophage interaction at in vitro scale, THP-1 monocytes were co-cultured with ADPGK KO or WT Ramos cells and their polarisation into M1/M2 macrophages was studied. In the setup, Ramos wild type or ADPGK KO cells were seeded on 0.4 µm Transwell inserts at a density of 1 million cells/ml. The cells in inserts were stimulated with PMA and moved into wells of 6-well plate containing 24 h PMA stimulated monocytes seeded at 200,000 cells/well in 2.5 ml of complete media. Cells were physically separated from each other, and migration was not possible due to small pore size of inserts, but the design of the setup permitted free sharing of media and soluble factors/nutrients between the cells. After 48 h of co-culture, macrophages were collected and analysed for M1/M2 markers of polarisation (hexokinase, Il-8, iNOS1 and Arg1) by RT-qPCR. Stimulated macrophages, without insert in co-culture, were used as control.

### Zebrafish xenograft studies

Zebrafish lines care and breeding were done under standardized and controlled conditions. Zebrafish *kdrl:GFP* line, used for xenografting, was raised at 28 °C in a dedicated Zebrafish facility available at our institute. Embryos obtained via in-cross matings were maintained in E3 Embryo medium. Twenty-three hours post fertilization (hpf), media was supplemented with 0.2 mM 1-phenyl-2- thiourea (PTU, Sigma-Aldrich), to prevent pigmentation of larvae. For xenotransplantation, Ramos WT and ADPGK KO cells were counted with a haemocytometer and stimulated with PMA. For injection, cells were fluorescently labelled by incubation with Cell Tracker CM-diI dye (C7001, Thermo Fisher) for 5 min at 37 °C and further 15 min at 4 °C. After the incubation, cells were washed once with PBS, and resuspended to a final concentration of 1.0 × 10^8^ cell/ml in RPMI. Zebrafish larvae, 48 hpf, were anesthetised with tricaine (0.02%, 168 mg/L, Sigma) and aligned on agarose moulds (1% agarose) in a lateral position. Around 200–250 CM-diI labelled cells were injected into the yolk sac of each zebrafish larva using glass microinjection needles (World Precision instruments, USA) with a FemtoJet microinjector (Eppendorf, Hamburg, Germany). Maximum pressure applied in the microinjector was 180 psi. Larvae were incubated at 28° C for 1 h and then transferred to 34 °C incubator for observing the growth of tumour cells.

Xenografts were analysed by fluorescence microscopy (Leica DMI 4000B, Leica microsystems) on the day of injection and 48 h post injection. Larvae were transferred to individual wells in 96-well plates in fresh E3 Embryo medium supplemented with PTU, for observation of tumour growth. At the time of microscopy, the E3 media was removed and larvae were embedded in freshly prepared 0.1% Agarose in embryo medium. Tricaine was added at a final concentration of 168 mg/L to anaesthetise the larvae. The low agarose concentration prevented movement of larvae during microscopy and assisted in their proper alignment and at the same time kept the larvae alive by keeping the fluid circulation intact. At the end of microscopy, larvae were resuspended in fresh E3 media. The embryo medium was replaced daily. Growth of xenografted cells in the larvae was assessed with the help of ImageJ software.

### Statistical analysis

Statistical analysis for all data presented in the study was performed using GraphPad Prism (7.04). Welch’s t-test (unpaired) was used for significance analysis. A p value less than 0.05 was considered significant. Two WT and two ADPGK KO cell lines were used for each experiment and their respective means (represented as WT and KO) were taken for final data analysis and representation after three independent experiments with each cell line. Unstimulated WT cells served as reference in all experiments, if not stated otherwise. Analysed data was presented as mean ± s.e.m of at least three independent experiments.

### Ethical statement

All the experiments involving Zebrafish were performed under approval of the ethics committee of the Medical Faculty of the University of Heidelberg (DIN EN ISO 9,001). Zebrafish housing and experiments were performed in accordance with all international and national laws and obligations as registered at the Regierungspräsidium Karlsruhe (Az. 35–9,185.81/G-85/16).

## Supplementary information

Supplementary Information

## Data Availability

All relevant data generated or analysed during this study are included in this published article. The datasets used and/or analysed during the current study but not available in the manuscript text are available from the corresponding author on reasonable request.
