# Supplementary material for: ADP-dependent glucokinase as a novel onco-target for haematological malignancies
Source: Sci Rep. 2020 Aug 12;10:13584. doi: 10.1038/s41598-020-70014-0 (PMC7423609; doi:10.1038/s41598-020-70014-0)
Supplement: Supplementary file 1 — Supplementary Information [file 41598_2020_70014_MOESM1_ESM.pdf]

ADP-dependent glucokinase as a novel onco-target for haematological malignancies

-Supplementary Information-

Tandon Amol[1#], Birkenhagen Jana[1], Nagalla Deepthi[2], Kölker Stefan[1], Sauer Sven Wolfgang[1]

# corresponding author

1. Division of Child Neurology and Metabolic Diseases, University Children's Hospital Heidelberg, Im Neuenheimer Feld 430, D-69120, Heidelberg, Germany.

2. German Cancer Research Center (DKFZ), 69120 Heidelberg, Germany.

Contact details of corresponding author:

1. Amol Tandon

Email: amoltandon@outlook.com,atandon@uab.edu

Phone: +1 205 2235337, +49 151 63602339

Present address: Department of Biochemistry and Molecular Genetics
University of Alabama at Birmingham
Birmingham, AL
35233
United States

Supplementary Table 1

Primer Sequences

Name	Sequence
18s Forward	GTAACCCGTTGAACCCCATT
18s Reverse	CCATCCAATCGGTAGTAGCG
ADPGK exon-2 Forward	GCTTCTTCCAGATCATTCCTTGA
ADPGK exon-2 Reverse	TTCAGGTTTCAGACCTACTTCCT
ADPGK CRISPR/Cas9 guide RNA	GTCAATGCATGTGTTGATGTGG
AID Forward	ATGGACAGCCTCTTGATGAAC
AID Reverse	CTCGTAAGTCATCAACCTCATACA
Arginase-1 Forward	ACTTAAAGAACAAGAGTGTGATGTG
Arginase-1 Reverse	GCATCCACCCAGATGACTCC
CDKN1A Forward	AGGTGGACCTGGAGACTCTCA
CDKN1A Reverse	CCTCTTGGAGAAGATCAGCCG
Hexokinase-2 Forward	CAAAGTGACAGTGGGTGTGG
Hexokinase-2 Reverse	GCCAGGTCCTTCACTGTCTC
Il-8 Forward	GAATGGGTTTGCTAGAATGTGATA
Il-8 Reverse	CAGACTAGGGTTGCCAGATTTAAC
iNOS Forward	GTCCCGAAGTTCTCAAGGCA
iNOS Reverse	CTGTGTCACTGGACTGGAGG
c-Myc proto oncogene Forward	AAAGGCCCCCAAGGTAGTTA
c-Myc proto oncogene Reverse	GCACAAGAGTTCCGTAGCTG
Translocated MYC Forward	CACTTTGCACTGGAACTTACAACA
Translocated MYC Reverse	TCACCATGTCTCCTCCCAGCA
XBP-1 unspliced Forward	GGAGTTAAGACAGCGCTTGG

XBP-1 unspliced Reverse	CTGCAGAGGTGCACGTAGTC
XBP-1 spliced Forward	CTGAGTCCGCAGCAGGTGCAG
XBP-1 spliced Reverse	ATCCATGGGGAGATGTTCTGG

Supplementary Table 2

<u>Antibodies</u>

Name	Company
CD20-PE	BD Biosciences, U.S.A.
CD138-PE	BD Biosciences, U.S.A.
β-Actin	Santa Cruz, U.S.A.
ADPGK	Sigma Aldrich, U.S.A.
AnnexinV-FITC	BD Biosciences, U.S.A.
Calreticulin	Abcam, Cambridge, UK
GFAT-2	Santa Cruz, U.S.A.
MYC proto-oncogene	Abcam, Cambridge, UK
PDI	Cell Signaling Technologies, U.S.A.
Cleaved Caspase-3	Cell Signaling Technologies, U.S.A.
Secondary, chicken anti-rabbit	Abcam, Cambridge, UK
Secondary, chicken anti-mouse	Abcam, Cambridge, UK

Supplementary Table 3

<u>Differences between Ramos WT and ADPGK KO MYC sequences</u>

	Ramos WT	ADPGK KO
No. of sequences analysed	30	30
Independent MYC mutations detected	38	10
Mutations after PMA stimulation	30	5

Mutations detected at D7	21	2
Mutations at AGC triplets	18	2
Mutations at GC sites	36	7

Supplementary data- MYC sequences (18/60) of Ramos WT and ADPGK KO used for analysis of mutations

>Ramos WT-1

GATGCATGCTCGAGCGGCCGCCAGTGTGATGGATATCTGCAGAATTCGCCCTTCACTTTGCACTGGAACTTACAAC
ACCCGACCAAGGACGCGACTCTCCCGACGCGGGGAGCCTATTGACATGCGTCGAATGACTACATAGGGTGTCTTTT
CTCTCACTCCTGCGACATCGACACTTTTCTCAGAGTAGGTTTGCAGCCTCCCGCGACGATGTCCCTCAACTTTACCTT
CACCAAGAAGAGCCATGACCTGGACTACGACTCGCTGCAGCCGCCGTATTTCTACTGCGACGAGGACGAGAACTTC
TACCAACAACAACAGCAGAGCGACCTGCAGCCCCCGGCGCCCAGCGAGGATATCTGGAAGAAATTCGAGCTGCTG
CCCATTCCGCCCCTGTCCCCTAGCCGCCGCTCCGGGCTCTGCTCGCCCTCCTACGTTGCGGTCACACCCTTCTCCCTT
CGGGGAGACAACGACGACGGTGGCGGGAGCTTCTCCACGGCCGACCAACTGCAGATGGTGACCGAGCTGCTGGG
AGGAGACATGGTGAAAGGGCGAATTCCAGCACACTGGCGGCCGTTACTAGTGGATCCGAGCTCGGTACCAAGCTT
GATGCATAGCTTGAGTATTCTATAGTGTCACCTAAATAGCTTGGCGTAATCATGGTCATAGCTGTTTCCTGTGTGAA
ATTGTTATCCGCTCACAATTCCACACAACATACGAGCCGGAAGCATAAAGTGTAAAGCCTGGGGTGCCTAATGAGT
GAGCTAACTCACATTAATTGCGTTGCGCTCACTGCCCGCTTTCCAGTCGGGAAACCTGTCGTGCCAGCTGCATTAAT
GAATCGGCCAACGCGCGGGGAGAGGCGGTTTGCGTATTGGGCGCTCTTCCGCTTCCTCGCTCACTGACTCGCTGCG
CTCGGTCGTTCGGCTGCGGCGAGCGGTATCAGCTCACTCAAAGGCGGTAATACGGTTATCCACAGAATCAGGGGA
TAACGCAGGAAAGAACATGTGAGCAAAAGGCCAGCAAAAGGCCAGGAACCGTAAAAAGGCCGCGTTGCTGGCGT
TTTTCCATAGGCTCCGCCCCCCTGACGAA

>Ramos WT-2

AAGCTATGCATCAAGCTTGGTACCGAGCTCGGATCCACTAGTAACGGCCGCCAGTGTGCTGGAATTCGCCCTTCAC
TTTGCACTGGAACTTACAACACCCGACCAAGGACGCGACTCTCCCGACGCGGGGAGCCTATTGACATGCGTCGAAT
GACTACATAGGGTGTCTTTTCTCTCACTCCTGCGACATCGACACTTTTCTCAGAGTAGGTTTGCAGCCTCCCGCGGC
GATGTCCCTCAACTTTACCTTCACCAAGAAGAGCCATGACCTGGACTACGACTCGCTACAGCCGCCGTATTTCTACT
GCGACGAGGACGAGAACTTCTACCAACAACAACAGCAGAGCGACCTGCAGCCCCCGGCGCCCAGCGAGGATATCT
GGAAGAAATTCGAGCTGCTGCCCATTCCGCCCCTGTCCCCTAGCCGCCGCTCCGGGCTCTGCTCGCCCTCCTACGTT
GCGGTCACACCCTTCTCCCTTCGGGGAGACAACGACGACGGTGGCGGGAGCTTCTCCACGGCCGACCAACTGGAG
ATGGTGACCGAGCTGCTGGGAGGAGACATGGTGAAAGGGCGAATTCTGCAGATATCCATCACACTGGCGGCCGCT
CGAGCATGCATCTAGAGGGCCCAATTCGCCCTATAGTGAGTCGTATTACAATTCACTGGCCGTCGTTTTACAACGTC
GTGACTGGGAAAACCCTGGCGTTACCCAACTTAATCGCCTTGCAGCACATCCCCCTTTCGCCAGCTGGCGTAATAGC
GAAGAGGCCCGCACCGATCGCCCTTCCCAACAGTTGCGCAGCCTATACGTACGGCAGTTTAAGGTTTACACCTATA
AAAGAGAGAGAGCCGTTATCGTCTGTTTGTGGATGTACAGAGTGATATTATTGACACGCCGGGGCGACGGATGGTGA
TCCCCCTGGCCAGTGCACGTCTGCTGTCAGATAAAGTCTCCCGTGAACTTTACCCGGTGGTGCATATCGGGGGATG
AAAGCTGGCGCATGATGACCACCGATATGGCCAGTGTGCCGGTCTCCGTTATCGGGGGAAGAAGTGGCTG

>ADPGK KO-1

CCTCTAGATGCATGCTCGAGCGGCCGCCAGTGTGATGGATATCTGCAGAATTCGCCCTTTCACCATGTCTCCTCCCA
GCAACTCGGTCACCATCTCCAGTTGGTCGGCCGTGGAGAAGCTCCCGCCACCGTCGTCGTTGTCTCCCCGAAGGGA

GAAGGGTGTGACCGCAACGTAGGAGGGCGAGCAGAGCCCGGAGCGGCGGCTAGGGGACAGGGGCGGAATGGG
CAGCAGCTCGAATTTCTTCCAGATATCCTCGCTGGGCGCCGGGGGCTGCAGGTCGCTCTGATGTTGTTGTTGGTAG
AAGTTCTCGTCCTCGTCGCAGTAGAAATACGGCGGCTGCAGCGAGTCGTAGTCCAGGTCATGGCTCTTCTTGGTGA
AGGTAAAGTTGAGGGACATCGTCGCGGGAGGCTGCAAACCTACTCTGAGAAAAGTGTCGATGTCGCAGGAGTGA
GAGAAAAGACACCCTATGTAGTCATTCGACGCATGTCAATAGGCTCCCCGCGTCGGGAGAGTCGCGTCCTTGGTCG
GGTGTTGTAAGTTCCAGTGCAAAGTGAAGGGCGAATTCCAGCACACTGGCGGCCGTTACTAGTGGATCCGAGCTC
GGTACCAAGCTTGATGCATAGCTTGAGTATTCTATAGTGTCACCTAAATAGCTTGGCGTAATCATGGTCATAGCTGT
TTCCTGTGTGAAATTGTTATCCGCTCACAATTCCACACAACATACGAGCCGGAAGCATAAAGTGTAAAGCCTGGGG
TGCCTAATGAGTGAGCTAACTCACATTAATTGCGTTGCGCTCACTGCCCGCTTTCCAGTCGGGAAACCTGTCGTGCC
AGCTGCATTAATGAATCGGCCAACGCGCGGGGAGAGGCGGTTTGCGTATTGGGCGCTCTTCCGCTTCCTCGCTCAC
TGACTCGCTGCGCTCGGTCGTTCGGCTGCGGCGAGCGGTATCAGCTCACTCAAAGGCGGTAATACGGTTATCCACA
GAATCAGGGGATAACGCAGGAAAGAACATGTGAGCAAAAGGCCAGCAAAAGGCCAGGAACCGTAAAAAGGCCG
CGTTGCTGGCGTTTTTCCATAGGCTCCGCCCCCCTGACGAGCATCA

>ADPGK KO-2

TNTGCATCAAGCTTGGTACCGAGCTCGGATCCACTAGTAACGGCCGCCAGTGTGCTGGAATTCGCCCTTCACTTTGC
ACTGGAACTTACAACACCCGACCAAGGACGCGACTCTCCCGACGCGGGGAGCCTATTGACATGCGTCGAATGACT
ACATAGGGTGTCTTTTCTCTCACTCCTGCGACATCGACACTTTTCTCAGAGTAGGTTTGCAGCCTCCCGCGACGATG
TCCCTCAACTTTACCTTCACCAAGAAGAGCCATGACCTGGACTACGACTCGCTGCAGCCGCCGTATTTCTACTGCGA
CGAGGACGAGAACTTCTACCAACAACAACAGCAGAGCGACCTGCAGCCCCCGGCGCCCAGCGAGGATATCTGGAA
GAAATTCGAGCTGCTGCCCATTCCGCCCCTGTCCCCTAGCCGCCGCTCCGGGCTCTGCTCGCCCTCCTACGTTGCGG
TCACACCCTTCTCCCTTCGGGGAGACAACGACGACGGTGGCGGGAGCTTCTCCACGGCCGACCAACTGGAGATGG
TGACCGAGTTGCTGGGAGGAGACATGGTGAAAGGGCGAATTCTGCAGATATCCATCACACTGGCGGCCGCTCGAG
CATGCATCTAGAGGGCCCAATTCGCCCTATAGTGAGTCGTATTACAATTCACTGGCCGTCGTTTTACAACGTCGTGA
CTGGGAAAACCCTGGCGTTACCCAACTTAATCGCCTTGCAGCACATCCCCCTTTCGCCAGCTGGCGTAATAGCGAA
GAGGCCCGCACCGATCGCCCTTCCCAACAGTTGCGCAGCCTATACGTACGGCAGTTTAAGGTTTACACCTATAAAA
GAGAGAGCCGTTATCGTCTGTTTGTGGATGTACAGAGTGATATTATTGACACGCCGGGGCGACGGATGGTGATCC
CCCTGGCCAGTGCACGTCTGCTGTCAGATAAAGTCTCCCGTGAACTTTACCCGGTGGTGCATATCGGGGGATGAAA
GCTGGCGCATGATGACCACCGATATGGCCAGTGTGCCGGTCTCCGTTATCGGGGAAGAAGTGGCTGATCTCAGCC
ACCG

>ADPGK KO-3

GGNCCTCTAGATGCATGCTCGAGCGGCCGCCAGTGTGATGGATATCTGCAGAATTCGCCCTTCACTTTGCACTGGA
ACTTACAACACCCGACCAAGGACGCGACTCTCCCGACGCGGGGAGCCTATTGACATGCGTCGAATGACTACATAG
GGTGTCTTTTCTCTCACTCCTGCGACATCGACACTTTTCTCAGAGTAGGTTTGCAGCCTCCCGCGACGATGTCCCTCA
ACTTTACCTTCACCAAGAAGAACCATGACCTGGACTACGACTCGCTGCAGCCGCCGTATTTCTACTGCGACGAGGA
CGAGAACTTCTACCAACAACAACAGCAGAGCGACCTGCAGCCCCCGGCGCCCAGCGAGGATATCTGGAAGAAATT
CGAGCTGCTGCCCATTCYGCCCCTGTCCCCTAGCCGCCGCTCCGGGCTCTGCTCGCCCTCCTACGTTGCGGTCACAC
CCTTCTCCCTTCGGGGAGACAACGACGACGGTGGCGGGAGCTTCTCCACGGCCGACCAACTGGAGATGGTGACCG
AGCTGCTGGGAGGAGACATGGTGAAAGGGCGAATTCCAGCACACTGGCGGCCGTTACTAGTGGATCCGAGCTCG
GTACCAAGCTTGATGCATAGCTTGAGTATTCTATAGTGTCACCTAAATAGCTTGGCGTAATCATGGTCATAGCTGTT
TCCTGTGTGAAATTGTTATCCGCTCACAATTCCACACAACATACGAGCCGGAAGCATAAAGTGTAAAGCCTGGGGT
GCCTAATGAGTGAGCTAACTCACATTAATTGCGTTGCGCTCACTGCCCGCTTTCCAGTCGGGAAACCTGTCGTGCCA
GCTGCATTAATGAATCGGCCAACGCGCGGGGAGAGGCGGTTTGCGTATTGGGCGCTCTTCCGCTTCCTCGCTCACT
GACTCGCTGCGCTCGGTCGTTCGGCTGCGGCGAGCGGTATCAGCTCACTCAAAGGCGGTAATACGGTTATCCACA

GAATCAGGGGATAACGCAGGAAAGAACATGTGAGCAAAAGGCCAGCAAAAGGCCAGGAACCGTAAAAAGGCCG
CGTTTGCTGGCGTTTTTCC

>ADPGK KO-4

CTNNAGCTATGCATCAAGCTTGGTACCGAGCTCGGATCCACTAGTAACGGCCGCCAGTGTGCTGGAATTCGCCCTT
CACTTTGCACTGGAACTTACAACACCCGACCAAGGACGCGACTCTCCCGACGCGGGGAGCCTATTGACATGCGTCG
AATGACTACATAGGGTGTCTTTTCTCTCACTCCTGCGACATCGACACTTTTCTCAGAGTAGGTTTGCAGCCTCCCGCG
ACGATGTCCCTCAACTTTACCTTCACCAAGAAGAGCCATGACCTGGACTACGACTCGCTGCAGCCGCCGTATTTCTA
CTGCGACGAGGACGAGAACTTCTACCAACAACAACAGCAGAGCGACCTGCAGCCCCCGGCGCCCAGCGAGGATAT
CTGGAAGAAATTCGAGCTGCTGCCCATTCCGCCCCTGTCCCCTAGCCGCCGCTCCGGGCTCTGCTCGCCCTCCTACG
TTGCGGTCACACCCTTCTCCCTTCGGGGAGACAACGACGACGGTGGCGGGAGCTTCTCCACGGCCGACCAACTGG
AGATGGTGACCGAGTTGCTGGGAGGAGACATGGTGAAAGGGCGAATTCTGCAGATATCCATCACACTGGCGGCC
GCTCGAGCATGCATCTAGAGGGCCCAATTCGCCCTATAGTGAGTCGTATTACAATTCACTGGCCGTCGTTTTACAAC
GTCGTGACTGGGAAAACCCTGGCGTTACCCAACTTAATCGCCTTGCAGCACATCCCCCTTTCGCCAGCTGGCGTAAT
AGCGAAGAGGCCCGCACCGATCGCCCTTCCCAACAGTTGCGCAGCCTATACGTACGGCAGTTTAAGGTTTACACCT
ATAAAAGAGAGAGCCGTTATCGTCTGTTTGTGGATGTACAGAGTGATATTATTGACACGCCGGGGCGACGGATGG
TGATCCCCCTGGCCAGTGCACGTCTGCTGTCAGATAAAGTCTCCCGTGAACTTTACCCGGTGGTGCATATCGGGGG
ATGAAAGCTGGCGCATGATGACCACCGATATGGCCAGTGTGCCCGGTCTCCGTTATCGGGGGAAGAAGNGGCT

>Ramos WT-D2-1

GGGCCCTCTAGATGCATGCTCGAGCGGCCGCCAGTGTGATGGATATCTGCAGAATTCGCCCTTCACTTTGCACTGG
AACTTACAACACCCGAGCAAGGACGCGACTCTCCCGACGCGGGGAGGCTATTCTGCCCATTTGGGGACACTTCCCC
GCCGCTGCCAGGACCCGCTTCTCTGAAAGGCTCTCCTTGCAGCTGCTTAGACGCTGGATTTTTTTCGGGTAGTGGAA
AACCAGCAGCCTCCCGCGACGATGCCCCTCAACGTTAGCTTCACCAACAGGAACTATGACCTCGACTACGACTCGG
TGCAGCCGTATTTCTACTGCGACGAGGAGGAGAACTTCTACCAGCAGCAGCAGCAGAGCGAGCTGCAGCCCCCGG
CGCCCAGCGAGGATATCTGGAAGAAATTCGAGCTGCTGCCCACCCCGCCCCTGTCCCCTAGCCGCCGCTCCGGGCT
CTGCTCGCCCTCCTACGTTGCGGTCACACCCTTCTCCCTTCGGGGAGACAACGACGGCGGTGGCGGGAGCTTCTCC
ACGGCCGACCAGCTGGAGATGGTGACCGAGCTGCTGGGAGGAGACATGGTGAAAGGGCGAATTCCAGCACACTG
GCGGCCGTTACTAGTGGATCCGAGCTCGGTACCAAGCTTGATGCATAGCTTGAGTATTCTATAGTGTCACCTAAAT
AGCTTGGCGTAATCATGGTCATAGCTGTTTCCTGTGTGAAATTGTTATCCGCTCACAATTCCACACAACATACGAGC
CGGAAGCATAAAGTGTAAAGCCTGGGGTGCCTAATGAGTGAGCTAACTCACATTAATTGCGTTGCGCTCACTGCCC
GCTTTCCAGTCGGGAAACCTGTCGTGCCAGCTGCATTAATGAATCGGCCAACGCGCGGGGAGAGGCGGTTTGCGT
ATTGGGCGCTCTTCCGCTTCCTCGCTCACTGACTCGCTGCGCTCGGTCGTTCGGCTGCGGCGAGCGGTATCAGCTCA
CTCAAAGGCGGTAATACGGTTATCCACAGAATCAGGGGATAACGCAGGAAAGAACATGTGAGCAAAAGGCCAGC
AAAAGGCCAGGAACCGTAAAAAGGCCGCGTTGCTGGCGTTTTTCCAAAGGGTC

>Ramos WT-D2-2

GCTGGAATTCGCCCTTCACTTTGCACTGGAACTTACAACACCCGACCAAGGACGCGACTCTCCCGACGCGGGGAGC
CTATTGACATGCGTCGAATGACTACATAGGGTGTCTTTTCTCTCACTCCTGCGCCATCGACACTTTTCTCAGAGTAGG
TTTGCAGCCTCCCGCGACGATGTCCCTCAACTTTACCTTCACCAAGAAGAGCCATGACCTGGACTACGACTCGCTGC
AGCCGTATTTCTACTGCGACGAGGACGAGAACTTCTACCAACAACAACAGCAGAGTGACCTGNAACCCCCGGCGC
CCAGCGAGGATATCTGGAAGAAATTCNAGCTGCTGCCCATTCCGCCCCTGTCCCCTAGCCGCCGCTCCGGGCTCTG
CTCGCCCTCCTACGTTGCGGTCACACCCTTCTCCCTTCGGGGAGACAACGACGACCGTGGCGGGAGCTTCTCCACG
GCCGACCAACTGGAGATGGTGACCGAGCTGCTGGGAGGAGACATGGTGAAAGGGCGAATTCTGCANATATCCAT
CACACTGGCGGCCGCTCGAGCATGCATCTAGAGGGCCCAATTCGCCCTATAGTGAGTCGTATTACAATTCACTGGC

CGTCGTTTTACAACGTCGTGACTGGGAAAACCCTGGCGTTACCCAACTTAATCGCCTTGCAGCACATCCCCCTTTCG
CCAGCTGGCG

>ADPGK KO-D2-1

TCTAGRTGCATGCTCGAGCGGCCGCCAGTGTGATGGATATCTGCAGAATTCGCCCTTTCACCATGTCTCCTCCCAGC
AACTCGGTCACCATCTCCAGTTGGTCGGCCGTGGAGAAGCTCCCGCCACCGTCGTCGTTGTCTCCCCGAAGGGAGA
AGGGTGTGACCGCAACGTAGGAGGGCGAGCAGAGCCCGGAGCGGCGGCTAGGGGACAGGGGCGGAATGGGCA
GCAGCTCGAATTTCTTCCAGATATCCTCGCTGGGCGCCGGGGGCTGCAGGTCGCTCTGCTGTTGTTGTTGGTAGAA
GTTCTCGTCCTCGTCGCAGTAGAAATACGGCGGCTGCAGCGAGTCGTAGTCCAGGTCATGGCTCTTCTTGGTGAAG
GTAAAGTTGAGGGACATCGTCGCGGGAGGCAAACCTACTCTGAGAAAAGTGTCGATGTCGCAGGAGTGAGAGAA
AAGACACCCTATGTAGTCATTCGACGCATGTCAATAGGCTCCCCGCGTCGGGAGAGTCGCGTCCTTGGTCGGGTGT
TGTAAGTTCCAGTGCAAAGTGAAGGGCGAATTCCAGCACACTGGCGGCCGTTACTAGTGGATCCGAGCTCGGTAC
CAAGCTTGATGCATAGCTTGAGTATTCTATAGTGTCACCTAAATAGCTTGGCGTAATCATGGTCATAGCTGTTTCCT
GTGTGAAATTGTTATCCGCTCACAATTCCACACAACATACGAGCCGGAAGCATAAAGTGTAAAGCCTGGGGTGCCT
AATGAGTGAGCTAACTCACATTAATTGCGTTGCGCTCACTGCCCGCTTTCCAGTCGGGAAACCTGTCGTGCCAGCTG
CATTAATGAATCGGCCAACGCGCGGGGAGAGGCGGTTTGCGTATTGGGCGCTCTTCCGCTTCCTCGCTCACTGACT
CGCTGCGCTCGGTCGTTCGGCTGCGGCGAGCGGTATCAGCTCACTCAAAGGCGGTAATACGGTTATCCACAGAATC
AGGGGATAACGCAGGAAAGAACATGTGAGCAAAAGGCCAGCAAAAGGCCAGGAACCGTAAAAAGGCCGCGTTG
CTGGCGTTTTTCCATAGGCTCCGCCCCCCTGACGAGCATCACA

>ADPGK KO-D2-2

ATCAAGCTTGGTACCGAGCTCGGATCCACTAGTAACGGCCGCCAGTGTGCTGGAATTCGCCCTTNNNTT

>ADPGK KO-D2-3

GATGCATGCTCGAGCGGCCGCCAGTGTGATGGATATCTGCAGAATTCGCCCTTCACTTTGCACTGGAACTTACAAC
ACCCGAGCAAGGACGCGACTCTCCCGACGCGGGGAGGCTATTCTGCCCATTTGGGGACACTTCCCCGCCGCTGCCA
GGACCCGCTTCTCTGAAAGGCTCTCCTTGCAGCTGCTTAGACGCTGGATTTTTTTCGGGTAGTGGAAAACCAGCAG
CCTCCCGCGACGATGCCCCTCAACGTTAGCTTCACCAACAGGAACTATGACCTCGACTACGACTCGGTGCAGCCGT
ATTTCTACTGCGACGAGGAGGAGAACTTCTACCAGCAGCAGCAGCAGAGCGAGCTGCAGCCCCCGGCGCCCAGCG
AGGATATCTGGAAGAAATTCGAGCTGCTGCCCACCCCGCCCCTGTCCCCTAGCCGCCGCTCCGGGCTCTGCTCGCC
CTCCTACGTTGCGGTCACACCCTTCTCCCTTCGGGGAGACAACGACGGCGGTGGCGGGAGCTTCTCCACGGCCGAC
CAGCTGGAGATGGTGACCGAGCTGCTGGGAGGAGACATGGTGAAAGGGCGAATTCCAGCACACTGGCGGCCGTT
ACTAGTGGATCCGAGCTCGGTACCAAGCTTGATGCATAGCTTGAGTATTCTATAGTGTCACCTAAATAGCTTGGCGT
AATCATGGTCATAGCTGTTTCCTGTGTGAAATTGTTATCCGCTCACAATTCCACACAACATACGAGCCGGAAGCATA
AAGTGTAAAGCCTGGGGTGCCTAATGAGTGAGCTAACTCACATTAATTGCGTTGCGCTCACTGCCCGCTTTCCAGTC
GGGAAACCTGTCGTGCCAGCTGCATTAATGAATCGGCCAACGCGCGGGGAGAGGCGGTTTGCGTATTGGGCGCTC
TTCCGCTTCCTCGCTCACTGACTCGCTGCGCTCGGTCGTTCGGCTGCGGCGAGCGGTATCAGCTCACTCACAGGCG

>ADPGK KO-D2-4

TCAAGCTATGCATCAAGCTTGGTACCGAGCTCGGATCCACTAGTAACGGCCGCCAGTGTGCTGGAATTCGCCCTTT
CACCATGTCTCCTCCCAGCAGCTCGGTCACCATCTCCAGCTGGTCGGCCGTGGAGAAGCTCCCGCCACCGCCGTCG
TTGTCTCCCCGAAGGGAGAAGGGTGTGACCGCAACGTAGGAGGGCGAGCAGAGCCCGGAGCGGCGGCTAGGGG
ACAGGGGCGGGGTGGGCAGCAGCTCGAATTTCTTCCAGATATCCTCGCTGGGCGCCGGGGGCTGCAGCTCGCTCT
GCTGCTGCTGCTGGTAGAAGTTCTCCTCCTCGTAGCAGTAGAAATACGGCTGCACCGAGTCGTAGTCGAGGTCATA
GTTCCTGTTGGTGAAGCTAACGTTGAGGGGCATCGTCGCGGGAGGCTGCTGGTTTTCCACTACCCGAAAAAAATCC

AGCGTCTAAGCAGCTGCAAGGAGAGCCTTTCAGAGAAGCGGGTCCTGGCAGCGGCGGGGAAGTGTCCCCAAATG
GGCAGAATAGCCTCCCCGCGTCGGGAGAGTCGCGTCCTTGCTCGGGTGTTGTAAGTTCCAGTGCAAAGTGAAGGG
CGAATTCTGCAGATATCCATCACACTGGCGGCCGCTCGAGCATGCATCTAGAGGGCCCAATTCGCCCTATAGTGAG
TCGTATTACAATTCACTGGCCGTCGTTTTACAACGTCGTGACTGGGAAAACCCTGGCGTTACCCAACTTAATCGCCT
TGCAGCACATCCCCCTTTCGCCAGCTGGCGTAATAGCGAAGAGGCCCGCACCGATCGCCCTTCCCAACAGTTGCGC
AGCCTATACGTACGGCAGTTTAAGGTTTACACCTATAAAAGAGAGAGCCGTTATCGTCTGTTTGTGGATGTACAGA
GTGATATTATTGACACGCCGGGGCGACGGATGGTGATCCCCCTGGCCAGTGCACGTCTGCTGTCAGATAAAGTCTC
CCGTGAACTTTACCCGGTGGTGCATATCGGGGATGAAAGCTGGCGCATGATGACCACCGATATGGCCAGTGTGCC
GGTCTCCGTTATCGGGGAAAGAAGTGGCTGATCTCAGC

>Ramos WT-D7-1

CTAGATGCATGCTCGAGCGGCCGCCAGTGTGATGGATATCTGCAGAATTCGCCCTTCACTTTGCACTGGAACTTAC
AACACCCGAGCAAGGACGCGACTCTCCCGACGCGGGGAGGCTATTCTGCCCATTTGGGGACACTTCCCCGCCGCT
GCCAGGACCCGCTTCTCTGAAAGGCTCTCCTTGCAGCTGCTTAGACGCTGGATTTTTTTCGGGTAGTGGAAAACCA
GCAGCCTCCCGCGACGATGCCCCTCAACGTTAGCTTCACCAACAGGAACTATGACCTCGACTACGACTCGGTGCAG
CCGTATTTCTACTGCGACGAGGAGGAGAACTTCTACCAGCAGCAGCAGCAGAGCGAGCTGCAGCCCCCGGCGCCC
AGCGAGGATATCTGGAAGAAATTCGAGCTGCTGCCCACCCCGCCCCTGTCCCCTAGCCGCCGCTCCGGGCTCTGCT
CGCCCTCCTACGTTGCGGTCACACCCTTCTCCCTTCGGGGAGACAACGACGGCGGTGGCGGGAGCTTCTCCACGGC
CGACCAGCTGGAGATGGTGACCGAGCTGCTGGGAGGAGACATGGTGAAAGGGCGAATTCCAGCACACTGGCGGC
CGTTACTAGTGGATCCGAGCTCGGTACCAAGCTTGATGCATAGCTTGAGTATTCTATAGTGTCACCTAAATAGCTTG
GCGTAATCATGGTCATAGCTGTTTCCTGTGTGAAATTGTTATCCGCTCACAATTCCACACAACATACGAGCCGGAAG
CATAAAGTGTAAAGCCTGGGGTGCCTAATGAGTGAGCTAACTCACATTAATTGCGTTGCGCTCACTGCCCGCTTTCC
AGTCGGGAAACCTGTCGTGCCAGCTGCATTAATGAATCGGCCAACGCGCGGGGAGAGGCGGTTTGCGTATTGGGC
GCTCTTCCGCTTCCTCGCTCACTGACTCGCTGCGCTCGGTCGTTCGGCTGCGGCGAGCGGTATCAGCTCACTCAAAG
GCMGGTA

>Ramos WT-D7-2

AGCTATGCATCAAGCTTGGTACCGAGCTCGGATCCACTAGTAACGGCCGCCAGTGTGCTGGAATTCGCCCTTCNTT
TTG

>ADPGK KO-D7-1

TNTAGATGCATGCTCGAGCGGCCGCCAGTGTGATGGATATCTGCAGAATTCGCCCTTCACTTTGCACTGGAACTTA
CAACACCCGAGCAAGGACGCGACTCTCCCGACGCGGGGAGGCTATTCTGCCCATTTGGGGACACTTCCCCGCCGCT
GCCAGGACCCGCTTCTCTGAAAGGCTCTCCTTGCAGCTGCTTAGACGCTGGATTTTTTTCGGGTAGTGGAAAACCA
GCCTCCCGCGACGATGCCCCTCAACGTTAGCTTCACCAACAGGAACTATGACCTCGACTACGACTCGGTGCAGCCG
TATTTCTACTGCGACGAGGAGGAGAACTTCTACCAGCAGCAGCAGCAGAGCGAGCTGCAGCCCCCGGCGCCCAGC
GAGGATATCTGGAAGAAATTCGAGCTGCTGCCCACCCCGCCCCTGTCCCCTAGCCGCCGCTCCGGGCTCTGCTCGC
CCTCCTACGTTGCGGTCACACCCTTCTCCCTTCGGGGAGACAACGACGGCGGTGGCGGGAGCTTCTCCACGGCCGA
CCAGCTGGAGATGGTGACCGAGCTGCTGGGAGGAGACATGGTGAAAGGGCGAATTCCAGCACACTGGCGGCCGT
TACTAGTGGATCCGAGCTCGGTACCAAGCTTGATGCATAGCTTGAGTATTCTATAGTGTCACCTAAATAGCTTGGCG
TAATCATGGTCATAGCTGTTTCCTGTGTGAAATTGTTATCCGCTCACAATTCCACACAACATACGAGCCGGAAGCAT
AAAGTGTAAAGCCTGGGGTGCCTAATGAGTGAGCTAACTCACATTAATTGCGTTGCGCTCACTGCCCGCTTTCCAG
TCGGGAAACCTGTCGTGCCAGCTGCATTAATGAATCGGCCAACGCGCGGGGAGAGGCGGTTTGCGTATTGGGCGC
TCTTCCGCTTCCTCGCTCACTGACTCGCTGCGCTCGGTCGTTCGGCTGCGGCGAGCGGTATCAGCTCACTCAAAGGC

GGTAATACGGTTATCCACAGAATCAGGGGATAACGCAGGAAAGAACATGTGAGCAAAAGGCCAGCAAAAGGCCA
GGAACCGTAAAAAGGCCG

>ADPGK KO-D7-2

ATACTCAAGCTATGCATCAAGCTTGGTACCGAGCTCGGATCCACTAGTAACGGCCGCCAGTGTGCTGGAATTCGCC
CTTCACTTTGCACTGGAACTTACAACACCCGACCAAGGACGCGACTCTCCCGACGCGGGGAGCCTATTGACATGCG
TCGAATGACTACATAGGGTGTCTTTTCTCTCACTCCTGCGACATCGACACTTTTCTCAGAGTAGGTTTGCCTCCCGCG
ACGATGTCCCTCAACTTTACCTTCACCAAGAAGAGCCATGACCTGGACTACGACTCGCTGCAGCCGCCGTATTTCTA
CTGCGACGAGGACGAGAACTTCTACCAACAACAACAGCAGAGCGACCTGCAGCCCCCGGCGCCCAGCGAGGATAT
CTGGAAGAAAATTCGAGCTGCTGCCCATTCCGCCCCTGTCCCCTAGCCGCCGCTCCGGGCTCTGCTCGCCCTCCTAC
GTTGCGGTCACACCCTTCTCCCTTCGGGGAGACAACGACGACGGTGGCGGGAGCTTCTCCACGGCCGACCAACTG
GAGATGGTGACCGAGTTGCTGGGAGGAGACATGGTGAAAGGGCGAATTCTGCAGATATCCATCACACTGGCGGC
CGCTCGAGCATGCATCTAGAGGGCCCAATTCGCCCTATAGTGAGTCGTATTACAATTCACTGGCCGTCGTTTTACAA
CGTCGTGACTGGGAAAACCCTGGCGTTACCCAACTTAATCGCCTTGCAGCACATCCCCCTTTCGCCAGCTGGCGTAA
TAGCGAAGAGGCCCGCACCGATCGCCCTTCCCAACAGTTGCGCAGCCTATACGTACGGCAGTTTAAGGTTTACACC
TATAAAAGAGAGAGCCGTTATCGTCTGTTTGTGGATGTACAGAGTGATATTATTGACACGCCGGGGCGACGGATG
GTGATCCCCCTGGCCAGTGCACGTCTGCTGTCAGATAAAGTCTCCCGTGAACTTTACCCGGTGGTGCATATCGGGG
GATGAAAGCTGGCGCATGATGACCACCGATATGGCCAGTGTGCCCGGTCTCCGTTATCGGGGGAAGAAGTGGCTG
A

>ADPGK KO-D7-3

AATTCNCCCTTGCTGGTGTTGCAGGAATGATAAACATTTTGCATGGTCTGGGCCTGCGTTTCCTTGCCCTTGGACAC
CACACACCATTTATGCATATTGGTATGGCACACAACTACAGAAATGTCAGCCAGGCCATCCAGGCAGTAAGGGATT
GTGGATATGAAATATCTTTAGGTCTGATGCCCAAGTCCATAGGCCCTCTTACATTTGTATTTACCGGCACCGGCAAC
GTGTCCAAGGGCGCTCAAGACATTTTCAACGAGCTCCCCTGTGAATTTGTGGAACCTCATGAACTGAAGGAAGTCT
CTTTGAGTGGAGACTTGACGAAAGTGTACGCCACAGTGATCAGCAGACATCACCACCTGATGAGGAGGAGTGACG
GACTCTATGACCCCCTGGAGTACGAAAACCACCCTGAGCTCTACACGTCACACTTTAGGGATACTGTGGCCCCTTAT
ACAACTTGTCTAATAAACGGTATATACTGGGACCCTCATACACCCAGACTGCTGAGGAGACTGGACGCCCAGCGGC
TGATTAGACCTGTTGTAACTACATCATCGTCAGCTGATTATGGCTGCCCTGCTCTTCCCCACAAGTTTTTGGCCATTT
GTGACATTTCTGCAGACACCGGTGGCTCCATTGAGTTCATGACTGAGTGTACAACTATTGAGAAACCTTTTTGTATG
TATGATGCCAACCAGCATATTGACCATGACAGTGTTGAAGGTAACGAAGGGCGAATTCCAGCACACTGGCGGCCG
TTACTAGTGGATCCGAGCTCGGTACCAAGCTTGATGCATAGCTTGAGTATTCTATAGCGTCACCTAAATAGCTTGGC
GTAATCATGGNCATAGCTGTRTCCTGWGTGAAATTGTTATCCGCTCRCAATTCCACACAACWTACGARCCGGAAG
CATAAAGTGTAAAGCCTGGGGTGCCTAATGARGGAACTAACTCAMATTAATTGCGGTGNNTCACTGCCCGCTTTCC
AGTCGGGAAACCTGTC

>ADPGK KO-D7-4

GCTATGCATCAAGCTTGGTACCGAGCTCGGATCCACTAGTAACGGCCGCCAGTGTGCTGGAATTCGCCCTTTCACC
ATGTCTCCTCCCAGCAGCTCGGTCACCATCTCCAGTTGGTCGGCCGTGGAGAAGCTCCCGCCACCGTCGTCGTTGTC
TCTCCGAAGGGAGAAGGGTGTGACCGCAACGTAGGAGGGCGAGCAGAGCCCGGAGCGGCGGCTAGGGGACAGG
GGCGGAATGGGCAGCAGCTCGAATTTCTTCCAGATATCCTCGCTGGGCGCCGGGGGCTGCAGGTCGCTCTGCTGT
TGTTGTTGGTAGAAGTTCTCGTCCTCGTCGCAGTAGAAATACGGCGGCTGCAGCGAGTCGTAGTCCAGGTCATGGC
TCTTCTTGGTGAAGGTAAAGTTGAGGGACATCGTCGCGGGAGGCTGCAAACCTACTCTGAGAAAAGTGTCGATGT
CGCAGGAGTGAGAGAAAAGACACCCTATGTAGTCATTCGACGCATGTCAATAGGCTCCCCGCGTCGGGAGAGTCG
CGTCCTTGGTCGGGTGTTGTAAGTTCCAGTGCAAAGTGAAGGGCGAATTCTGCAGATATCCATCACACTGGCGGCC

GCTCGAGCATGCATCTAGAGGGCCCAATTCGCCCTATAGTGAGTCGTATTACAATTCACTGGCCGTCGTTTTACAAC
GTCGTGACTGGGAAAACCCTGGCGTTACCCAACTTAATCGCCTTGCAGCACATCCCCCTTTCGCCAGCTGGCGTAAT
AGCGAAGAGGCCCGCACCGATCGCCCTTCCCAACAGTTGCGCAGCCTATACGTACGGCAGTTTAAGGTTTACACCT
ATAAAAGAGAGAGCCGTTATCGTCTGTTTGTGGATGTACAGAGTGATATTATTGACACGCCGGGGCGACGGATGG
TGATCCCCCTGGCCAGTGCACGTCTGCTGTCAGATAAAGTCTCCCGTGAACTTTACCCGGTGGTGCATATCGGGGG
ATGAAAGCTGGCGCATGATGACCACCGATATGGCCAGTGTGCCGGTCTCCGTTATCGGGGGAAGAAGTGGCTGAT
CTCAGCCACCGCGAAAATGACATCAAAAACGCCATTAAC

Supplementary Figure

Full Size Blot Images of Western blots used in manuscript

Figures in the manuscript are depicted in cropped formats, showing the protein of interest with size marker. We have provided here the full size blots along with protein ladders, for the figures used in manuscript, wherever possible. The protein ladder with size markers, used for each of the blots is provided at the end.

β-Actin, Fig. 1b

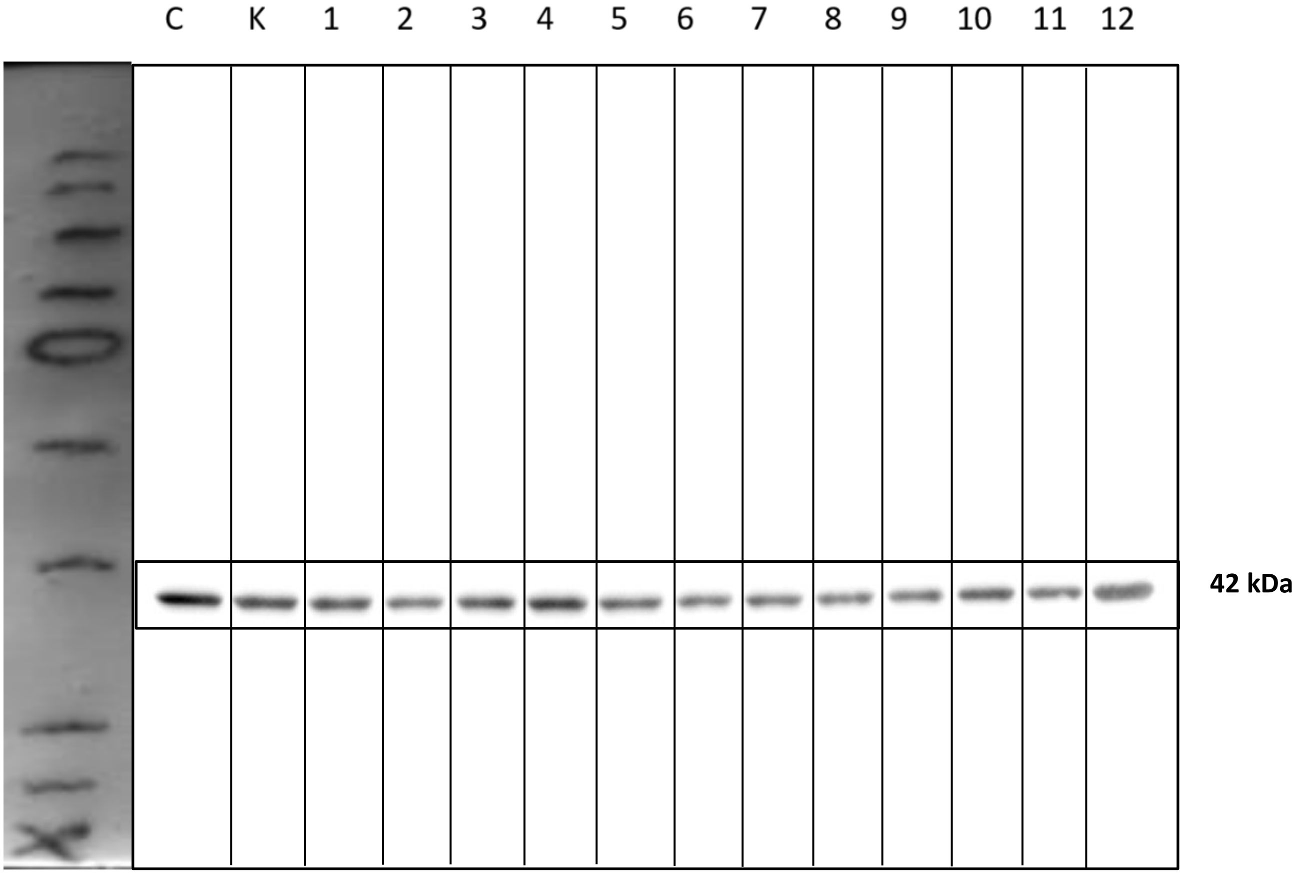

ADP-dependent glucokinase (ADPGK) – cleaved, 46 kDa protein is the band of interest, **Fig. 1b**

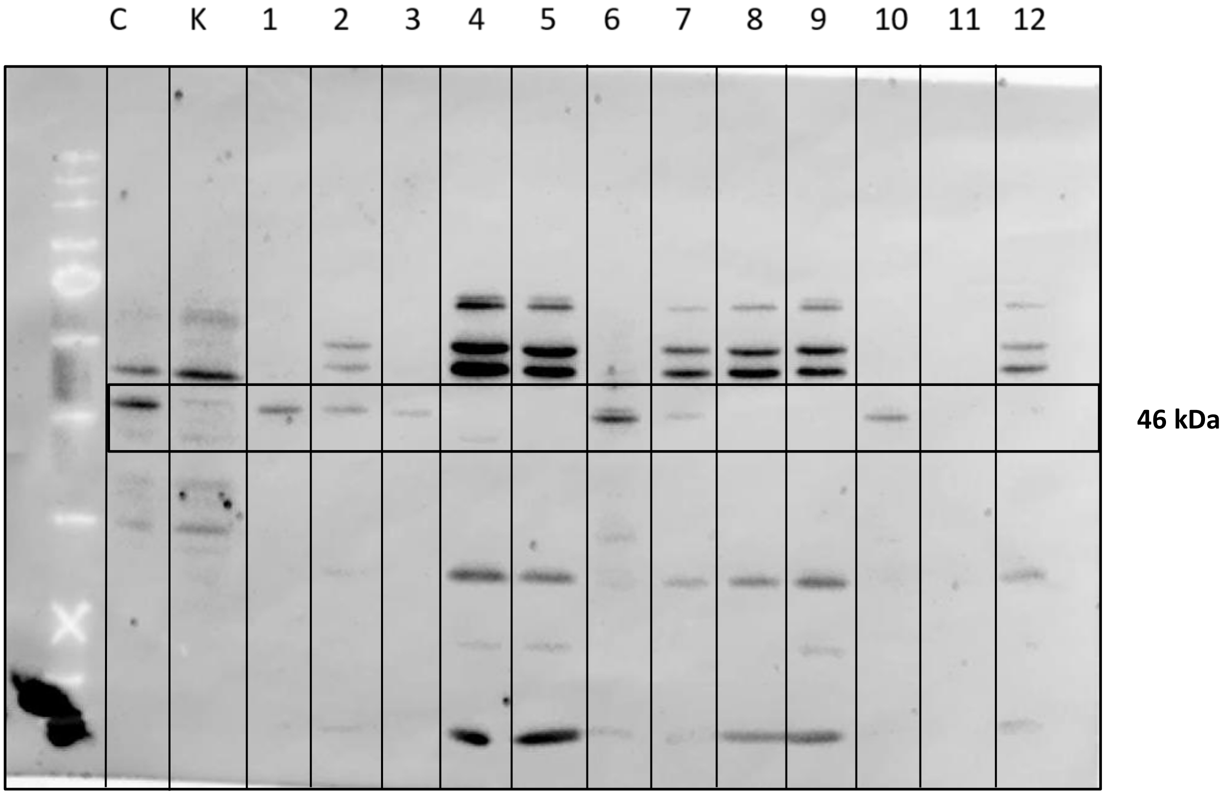

Caspase-3 Cleaved, Fig. 2f

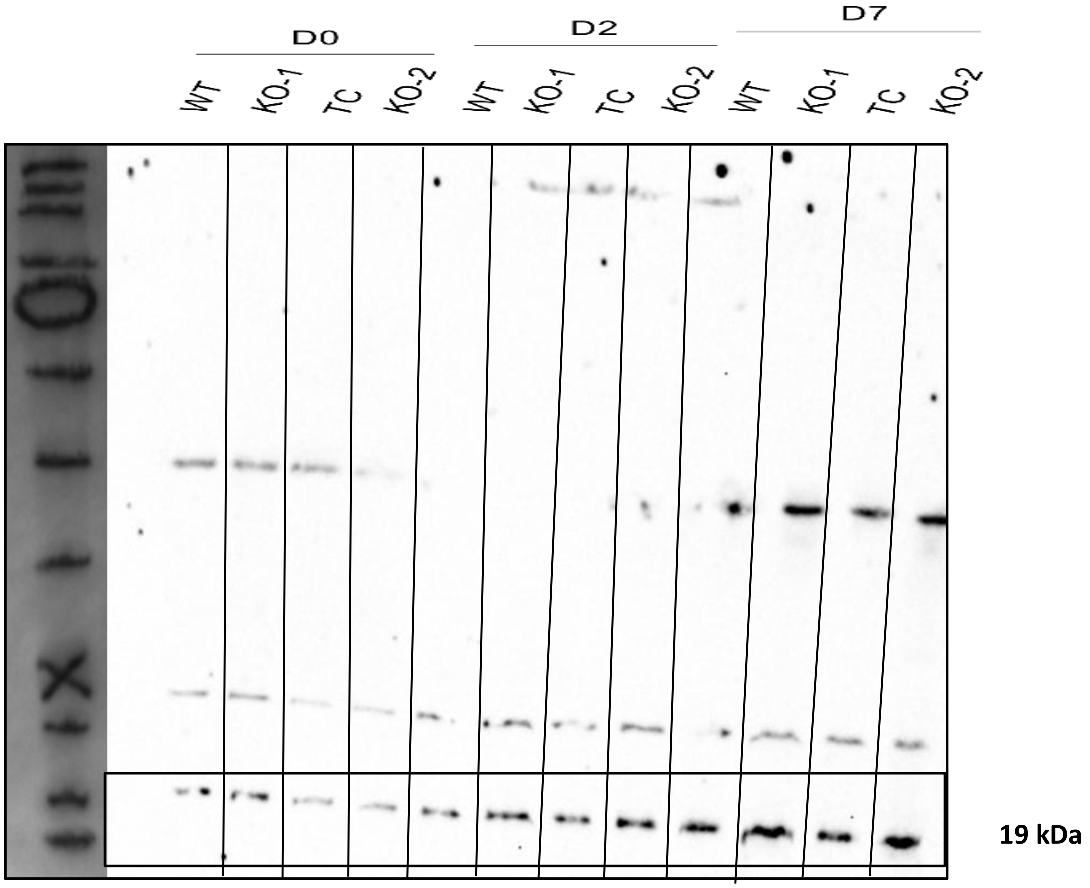

β-Actin, Fig. 2f

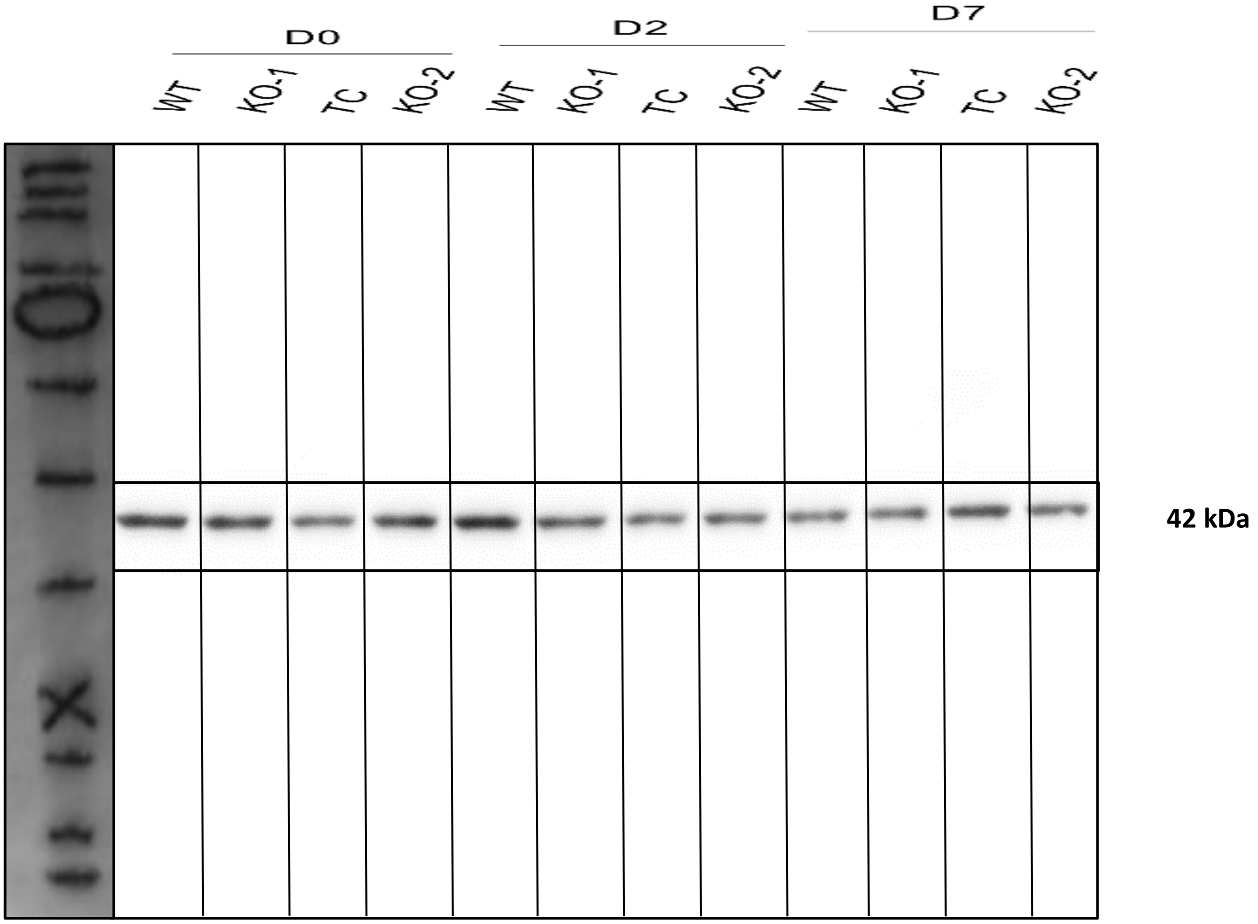

Protein Disulphide Isomerase (PDI), Fig. 3d

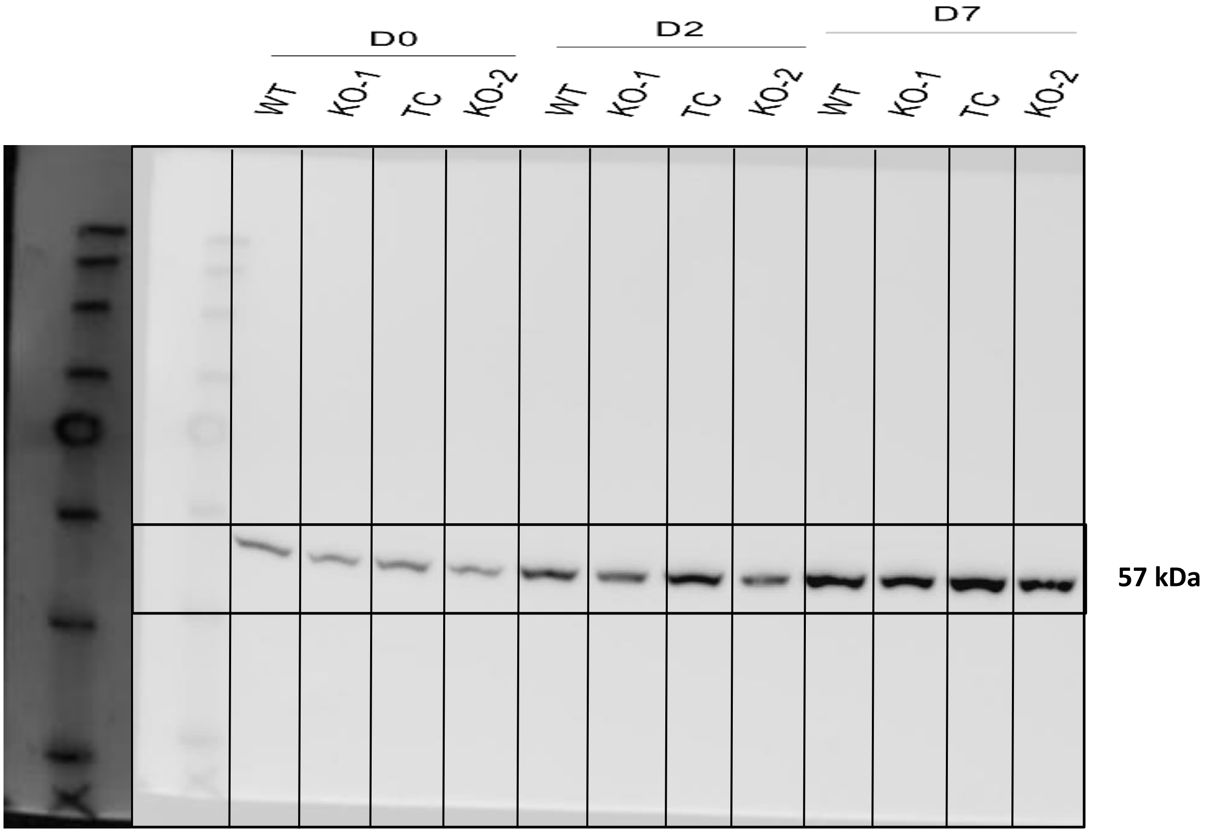

Calreticulin, Fig. 3d (Reprobed with β-Actin, 42 kDa)

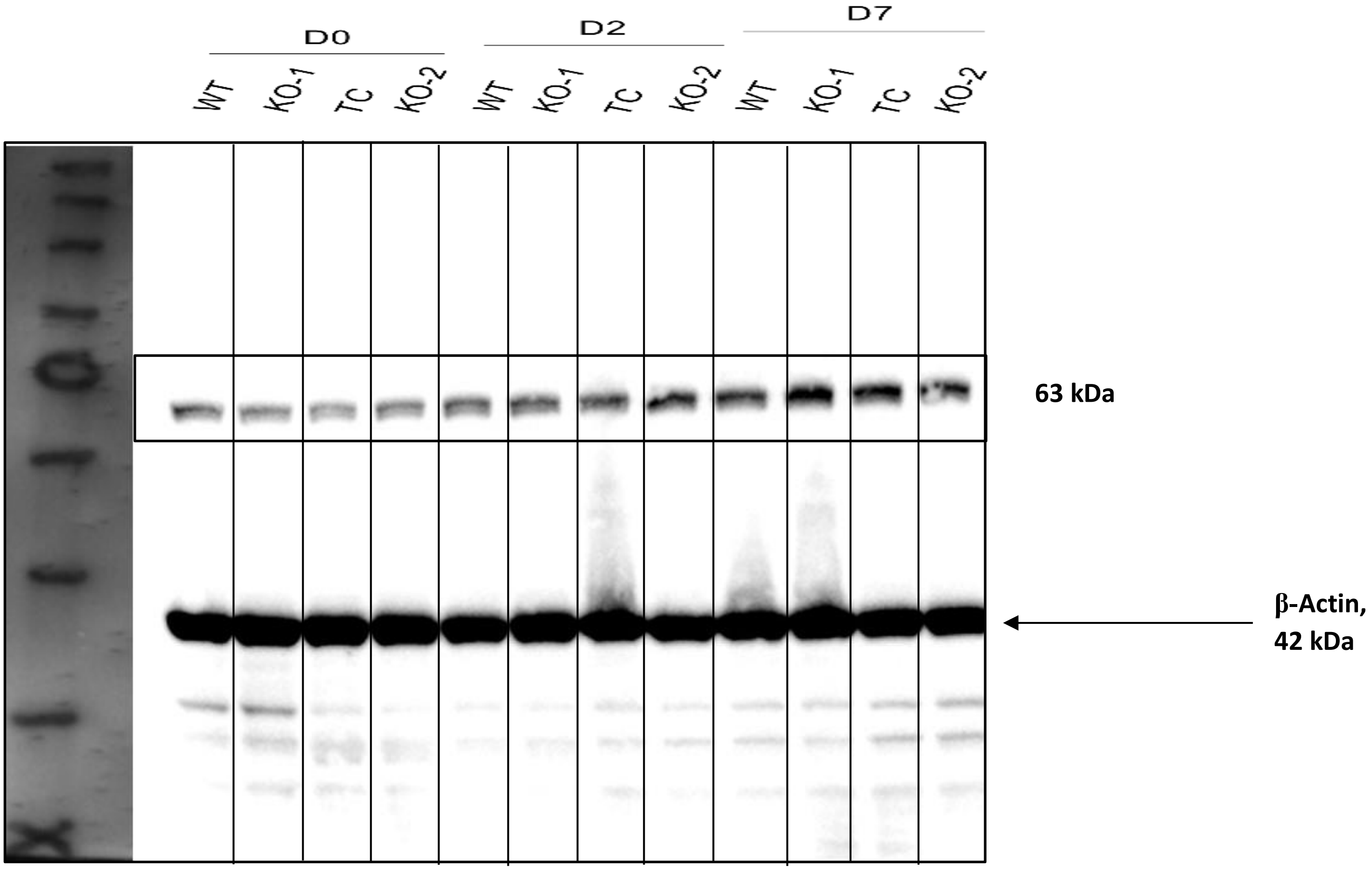

β-Actin, Fig. 3d

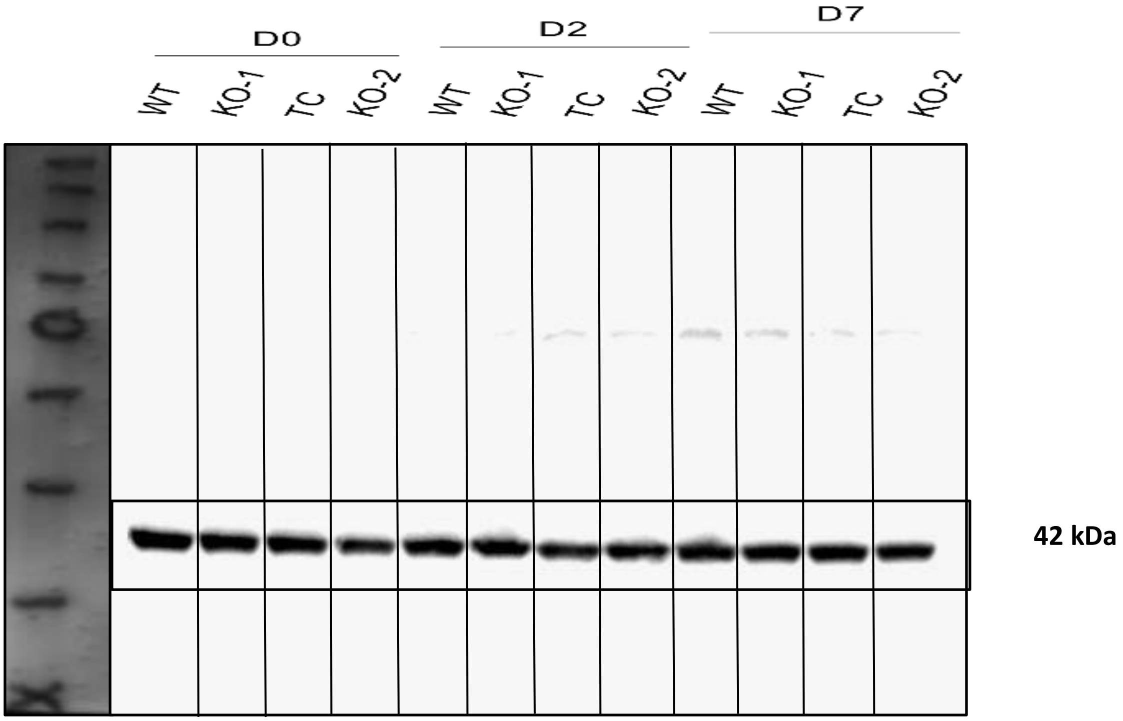

C-Myc, Fig. 5b

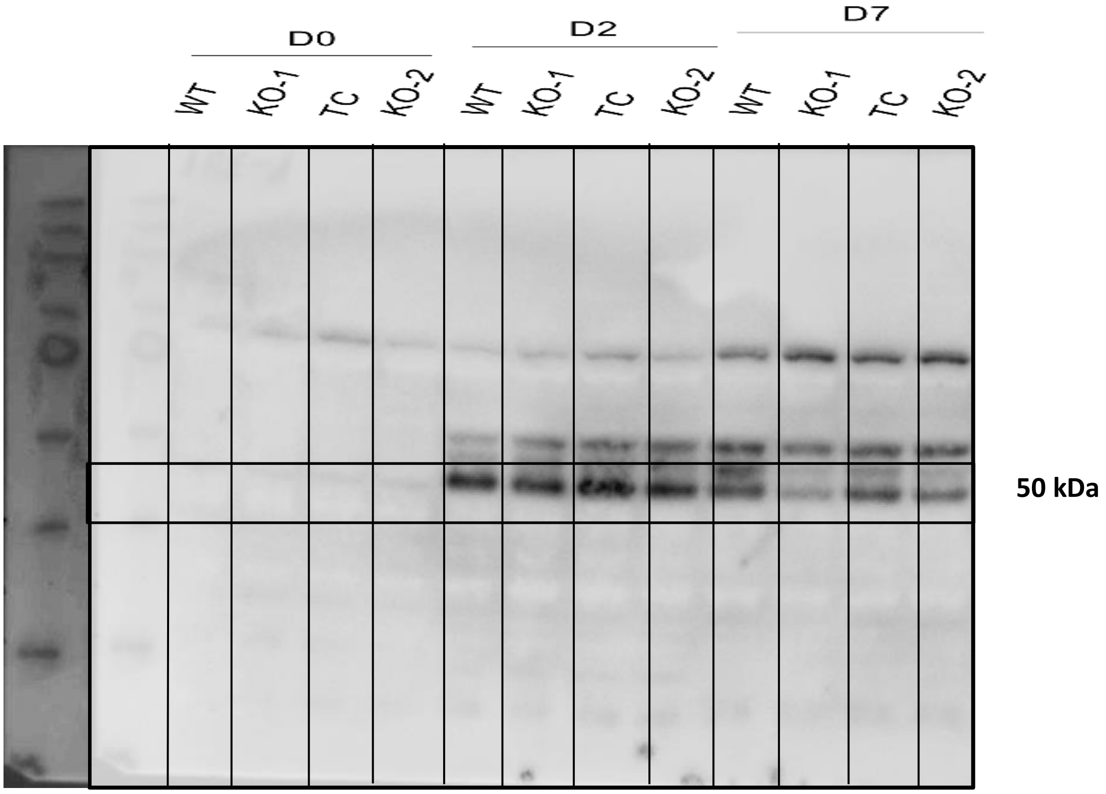

β-Actin, Fig. 5b

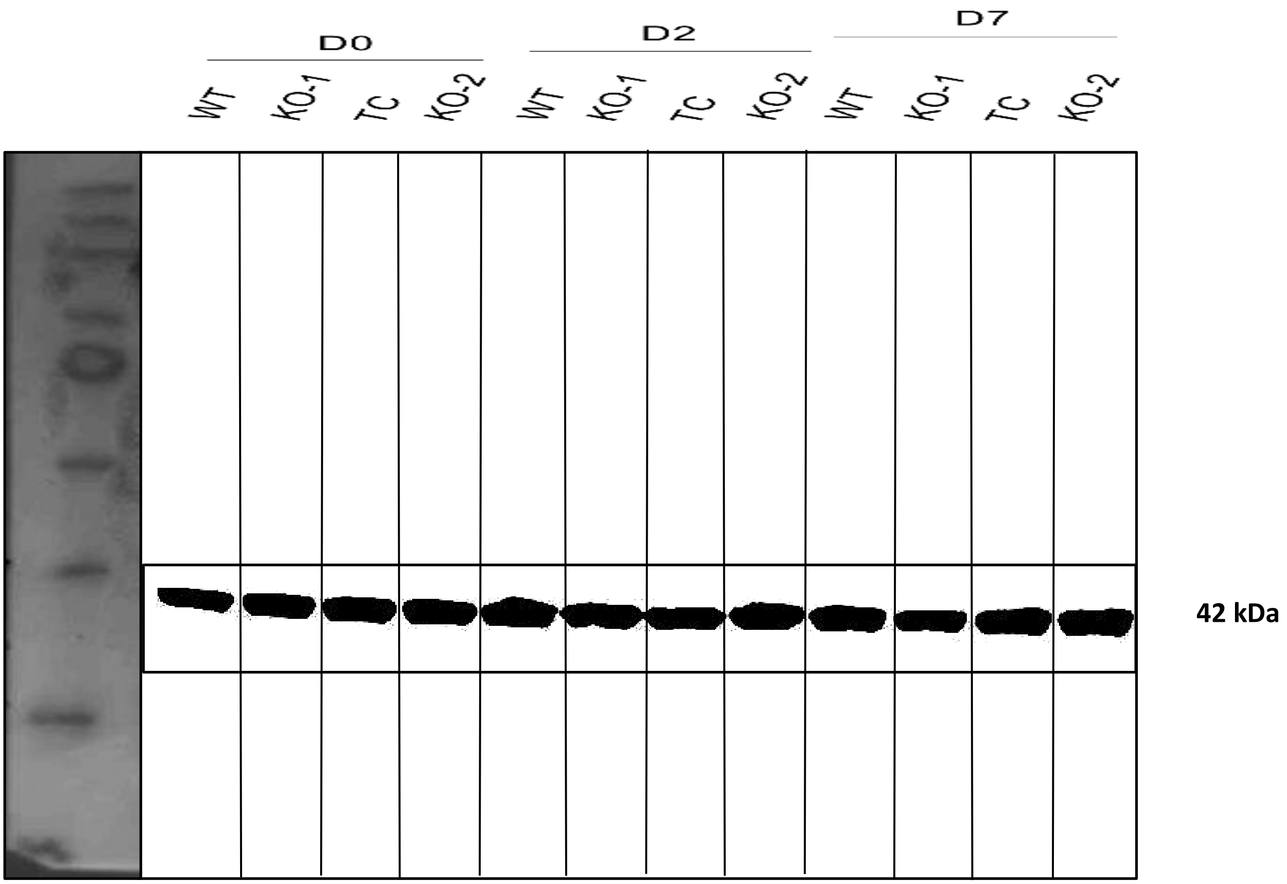

Protein size marker – NEB, Color Prestained Protein Standard, Broad Range (11–245 kDa), P7712

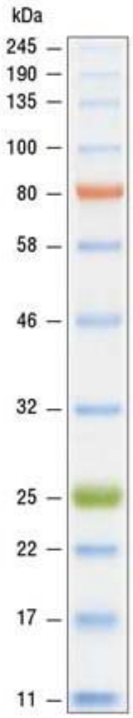
kDa
245
190
135
100
80
58
46
32
25
22
17
11
